# Self‐Processing Circuits Among Depressed Youth After Amygdala Neurofeedback Cued to the Self‐Face

**DOI:** 10.1002/jnr.70097

**Published:** 2025-12-12

**Authors:** Sewon Oh, Niki Hosseini‐Kamkar, Carmen Santana‐Gonzalez, Janani Ranatunga, Giang‐Hoang Nguyen, Matthew Maurice, Kymberly Young, Karina Quevedo

**Affiliations:** ^1^ Department of Psychology University of South Carolina Columbia South Carolina USA; ^2^ Institute for Mind and Brain University of South Carolina Columbia South Carolina USA; ^3^ Atlas Institute for Veterans and Families Ottawa Ontario Canada; ^4^ Rochester Institute of Technology Dubai Dubai UAE; ^5^ Division of Child and Adolescent Psychiatry, Department of Psychiatry and Behavioral Sciences University of Minnesota Minneapolis Minnesota USA; ^6^ Department of Psychiatry Western Psychiatric Institute and Clinic, School of Medicine, University of Pittsburgh Pittsburgh Pennsylvania USA

**Keywords:** adolescence, amygdala, cortical midline structures, depression, emotion regulation, neurofeedback, self‐face recognition, self‐processing

## Abstract

Major depressive disorder is a significant health concern among adolescents due to its link to suicide and lifetime impairments. Current treatments yield limited success, but real‐time functional magnetic resonance imaging (RT‐fMRI) neurofeedback (NF) is a promising intervention. We tested the effects of RT‐fMRI neurofeedback on the modulation of neural circuitry underlying self‐referential processing and emotion regulation in youth trying to up‐regulate the amygdala‐hippocampus (AMYHIPP) complex. We examined amygdala functional connectivity during a self‐other face recognition task before and after RT‐fMRI NF from the AMYHIPP complex. (1) Depressed youth showed higher bilateral amygdala to anterior cingulate cortex (ACC), superior temporal and frontal gyri connectivity compared to controls, who showed lower connectivity before NF. Yet after NF, this pattern reversed, with depressed youth showing lower bilateral amygdala connectivity than controls. (2) Depressed youth showed *increased right* amygdala‐cuneus connectivity while controls showed *increased left* amygdala‐cuneus connectivity during self‐other face recognition. (3) Lower right amygdala‐cuneus connectivity was linked to improved rumination. Higher left amygdala‐cuneus connectivity was linked to improved depression. (4) Shifts of less right versus left amygdala to superior middle temporal gyri connectivity after neurofeedback, and of more right versus left amygdala to middle frontal gyrus after NF, were linked to higher AMYHIPP engagement during training. This is suggestive of NF training effects upon implicit versus explicit emotion circuits. Caution is necessary regarding the meaning of symptoms to circuits associations and putative mechanisms of neurofeedback training given the lack of a placebo group.

## Significance Statement

Current depression treatments have moderate effectiveness and longevity. We address a public health need by testing an innovative neuroimaging treatment, real‐time fMRI neurofeedback, from the amygdala and hippocampus paired with self‐face in depressed adolescents. Posttreatment amygdala connectivity changes during self‐referential processing are linked to reduced emotion dysregulation and depression. Comparing this intervention with an active control will elucidate the effectiveness of this short neurofeedback treatment targeting self‐referential networks. Our findings open avenues for targeted interventions that modulate self‐processing neural networks. Further exploration of connectivity directionality and its clinical impact will enhance the significance of this line of research.

## Graphical abstract

RT‐fMRI neurofeedback targeting the amygdala‐hippocampus complex was followed by changes in amygdala connectivity with prefrontal, temporal, and occipital regions in depressed youth. Neurofeedback was also associated with hemisphere‐specific amygdala‐cuneus shifts that were associated with improvements in rumination and depression symptoms.

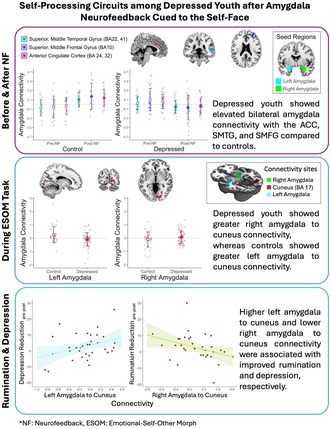

## 1 Introduction

Major depressive disorder (MDD) affects an estimated 300 million people worldwide (WHO 2017, NIMH 2023); furthermore, the overall incidence of depression has increased by approximately 49.8% from 1990 to 2017 making it a growing public health concern (Liu et al. 2020). Critically, the prevalence of depression peaks in youth aged 10–19 years, with 34% of adolescents globally at risk for developing clinical depression—a statistic that exceeds the risk for individuals aged 18–25 years (Shorey et al. 2022). Earlier onset of depression predicts symptom severity, comorbidities with other psychiatric disorders, and a greater likelihood of suicide attempts (Sung et al. 2013). These statistics demonstrate that adolescence is a sensitive developmental period for the onset of mental health disorders, including depression (Andersen and Teicher 2008; Richards 2011; Shorey et al. 2022). Considering the human suffering and public health burden linked to depression, it is imperative to develop treatments aimed at depression in adolescence. Currently available therapies, including medication and psychotherapy, are effective for some individuals, yet fail to prevent chronic depression and do not improve symptoms for all individuals (Al‐Harbi 2012; Wang et al. 2003). For example, 30%–50% of youth do not respond well to medications or cognitive behavioral therapy (Al‐Harbi 2012; Cheung et al. 2007; Vitiello et al. 2011), and some experience increased suicidal thoughts with serotonergic antidepressants (Reeves and Ladner 2010).

In the last two decades, real‐time functional magnetic resonance imaging (RT‐fMRI) neurofeedback has emerged as a promising standalone or supplemental treatment to existing therapies and an alternative for treatment‐resistant depression (Tschentscher et al. 2024). Neurofeedback is a noninvasive brain‐computer interface that can be used to train individuals to self‐regulate the activity of brain regions and networks. Indeed, RT‐fMRI neurofeedback has been used to improve symptoms in a broad spectrum of psychiatric conditions (Chiba et al. 2019; Gerin et al. 2016; Hamilton et al. 2016; Li et al. 2013; Lieberman et al. 2023; Linden et al. 2012; Mehler et al. 2018; Misaki et al. 2021, 2018, 2019; Nicholson et al. 2017, 2018; Okano et al. 2020; Scheinost et al. 2013; Schoenberg and David 2014; Sukhodolsky et al. 2020; Weaver et al. 2020; Young et al. 2017; Zhao et al. 2023; Zotev et al. 2013; Zweerings et al. 2018, 2020). One key question regarding the use of RT‐fMRI is the identification of neural structures and networks to be targeted and modulated. Adolescence is marked by substantial pruning (Ahmed et al. 2015; Sakai 2020) and specialization of cortico‐limbic circuits (Heller et al. 2016; Tottenham 2020), which coincides with the maturation of identity and selfhood (Ahmed et al. 2015; Klimstra et al. 2010), self‐processing (van Buuren et al. 2022), and emotion regulation abilities (Ahmed et al. 2015; Tottenham 2020). Consequently, interventions that modulate neural circuitry involved in self‐referential processing and emotion regulation may be effective in treating mental health disorders that develop during adolescence.

Self‐processing entails conscious and unconscious assimilation of information pertaining to one's identity. Examples of self‐processing include the identification and recognition of one's own facial features, as well as subjective evaluations of one's own traits and skills. A core depression symptom is negative self‐processing (i.e., feelings and cognitions of worthlessness and helplessness: i.e., the broken self). Thus, depression is partly rooted in negative self‐processing biases, which in children and adolescents predict deep‐held beliefs and MDD later in life (Butterfield et al. 2023; LeMoult et al. 2017; Victoria et al. 2019). These vulnerabilities of adolescence‐onset MDD occur along transformations typical of this period, such as heightened processing of negative self‐relevant stimuli in adolescent girls (Moses‐Payne et al. 2022) and changes in the neurocircuitry of emotion regulation (Rosenkranz 2024) and self‐processing.

The neural circuitry underlying self‐processing includes the prefrontal cortex (PFC), insula, anterior cingulate cortex (ACC), posterior cingulate cortex (PCC), precuneus, and superior temporal gyrus (STG) (Kircher et al. 2001; Northoff et al. 2006). The regions anatomically located along the midline, such as the ACC, PCC, and precuneus, are collectively referred to as cortical midline structures (CMS), function together with medial prefrontal regions like dorsomedial PFC (dmPFC) and orbitomedial PFC (omPFC) to support self‐referential processing (Northoff and Bermpohl 2004). Highly self‐relevant information engages neural mechanisms that are also recruited during voluntary self‐reflection; for example recognition of pictures of our face or our physical features recruits CMS structures (Quevedo et al. 2018).

CMS are differentially engaged by depressed adolescents (Bradley et al. 2016). Depressed youth show reduced connectivity between dmPFC, PCC, and precuneus when engaging in self‐referential appraisals of positive and negative traits (Bradley et al. 2016). This CMS hypo‐connectivity may reflect impaired coordination among regulatory cortical structures, potentially compromising higher‐order cognitive functions during self‐evaluation in depressed youth. By contrast, there is increased connectivity between the amygdala, ventromedial PFC, and subgenual ACC when depressed youth engage in rumination during self‐appraisals (Fowler et al. 2017; Murphy et al. 2016). Furthermore, higher amygdala to subgenual ACC connectivity is associated with increased negative affect, and these alterations in connectivity patterns serve as predictors for the onset of depression in adolescents (Davey et al. 2015). The latter is consistent with our work using the self‐other face recognition task (Emotional‐Self‐Other Morph; ESOM), at the core of this report. Specifically, we found that depressed youth show higher dorsolateral prefrontal cortex activity than controls (Quevedo et al. 2016), and that higher suicide risks are associated with higher left amygdala connectivity to anterior cingulate cortex (Alarcon et al. 2019) in depressed youth.

To summarize, reduced functional coordination among cortical regions, and heightened functional connectivity between the amygdala and CMS during self‐processing may characterize adolescent depression. Nevertheless, the specific pattern of hyper‐ versus hypo‐connectivity between the amygdala and cortical regions varies depending on context, such as whether resting state versus emotionally challenging tasks are used in neuroimaging paradigms. These differences across studies may help partially explain the divergent findings in the literature. Whether neurofeedback interventions can modify amygdala‐CMS connectivity during self‐processing tasks (e.g., recognizing one's face), is an open question that we begin to broach in this report.

The development of emotion regulation represents a critical milestone during adolescence. Emotion regulation is mediated by neural regions like the amygdala, and involves the management of affective experiences, including the processing of, and rapid behavioral responses to facial expressions (Besteher et al. 2020; Eisenberg and Spinrad 2004). Emotion regulation and self‐referential processing (e.g., self‐face recognition) share overlapping neural circuits, with the ACC playing a significant role in facilitating both processes (Eisenberg and Spinrad 2004; Heatherton et al. 2006; Hu et al. 2016; Kircher et al. 2001). The amygdala and hippocampus enable the retrieval of emotion‐laden memories by reactivating stored representations in the neocortex (Fenker et al. 2005). Together, they govern emotional experiences and affective recall (Campbell et al. 2018). These regions are reciprocally connected to the ACC and CMS to enable functions such as long‐term memory, self‐processing, and emotion regulation (Belzung et al. 2015; Eisenberg and Spinrad 2004; Kircher et al. 2001; Zotev et al. 2013). Notably, the connectivity between the amygdala and hippocampus (AMYHIPP) and AMYHIPP‐CMS strengthens during the recall of positive autobiographical memory in healthy adults (de Voogd et al. 2017; Nawa and Ando 2019). However, the circuitry involved in emotion regulation exhibits variability depending on diagnostic factors and the emotional valence of stimuli. For example, individuals with MDD display hypo‐connectivity between AMYHIPP and dmPFC, blunted fronto‐insular connectivity during resting states, and biased recall of negative autobiographical memories (Anand et al. 2009). Thus, the disrupted functional connectivity between these areas may serve as the underlying biological mechanism for both inappropriate emotional responses and impaired processing of affective memories in depressed individuals (Dore et al. 2018; Tahmasian et al. 2013). As animal models indicate significant corticolimbic connectivity changes during adolescence (Cunningham et al. 2002), and emerging hemispheric specialization during adolescence might make the AMYHIPP complex a malleable target for early noninvasive neuromodulation such as neurofeedback.

This study builds upon our previous research, which examined blood oxygen level dependent (BOLD) activity during neurofeedback and self‐referential processing (Quevedo et al. 2019), as well as amygdala connectivity during one neurofeedback day and its associations with symptoms' improvement (Quevedo et al. 2020). Considering the well‐documented disrupted functional connectivity between the AMYHIPP complex and CMS in depression (Dore et al. 2018; Hamilton and Gotlib 2008; Tahmasian et al. 2013), along with their involvement in self‐processing and emotion regulation (Campbell et al. 2018), the AMYHIPP complex was our selected target region for neurofeedback training. More importantly, Dr. Quevedo's work showed that depressed youth evidenced hypoactive mid‐temporal limbic activity (bilateral amygdala, hippocampus and fusiform) relative to controls when identifying their own happy faces versus neutral faces, yet showed hyperactivity in those areas when identifying other unfamiliar happy faces versus neutral faces, In contrast healthy youth showed the opposite pattern (Quevedo et al. 2018). Such results suggested disturbed emotional saliency, memory and enjoyment for positive self‐referential cues. A self‐serving bias (positive self‐referential behavior, reflexes and feelings about ourselves) is typical (and linked to wellbeing) among healthy individuals. To assuage the abnormal neurobiology of visual self‐processing noted in our work (Quevedo et al. 2018), our proposed neurofeedback training sought to enhance bilateral amygdala and hippocampus activity paired to the self‐happy face, to counter dampened visual self‐referential processing in depressed youth. Similarly, we sought to examine potential neural changes during self‐other face recognition (Emotional‐Self‐Other Morph; ESOM) task after neurofeedback training paired to the self‐face.

Here we examine AMYHIPP‐cortical connectivity during that self‐other face recognition task (Emotional‐Self‐Other Morph, ESOM) administered before and after neurofeedback cued to the self‐face, while also investigating the association between those circuit's functional connectivity and performance (levels of AMYHIPP activity) during the neurofeedback training. Specifically, we also tested whether amygdala circuits' functional connectivity during self‐processing alterations from pre‐ to post‐ neurofeedback were associated with performance (i.e., AMYHIPP activity levels) during a single neurofeedback session during which AMYHIPP modulation was paired to the self‐face.

Understanding connectivity changes after versus before neurofeedback training allows us to test whether the neuromodulating effects of neurofeedback persist beyond the training period, and may suggest modifications to the neurofeedback target to increase the success of the intervention and sustain neurofeedback learning effects. Note that amygdala activity and connectivity during neurofeedback and its links to symptoms' change training were already reported by our research group (Ahrweiler et al. 2022; Quevedo et al. 2019, 2020). In addition to elucidating the mechanisms underlying neurofeedback (NF) as a potential treatment for depressive disorders, examining differences in amygdala circuitry between diagnostic groups provides insights into whether NF can be utilized in a compensatory or remedial manner to modulate self‐ and emotion processing circuits, informing its potential use as a standalone treatment for depression or its use in conjunction with existing pharmacological and psychotherapeutic approaches.

**FIGURE 1:**
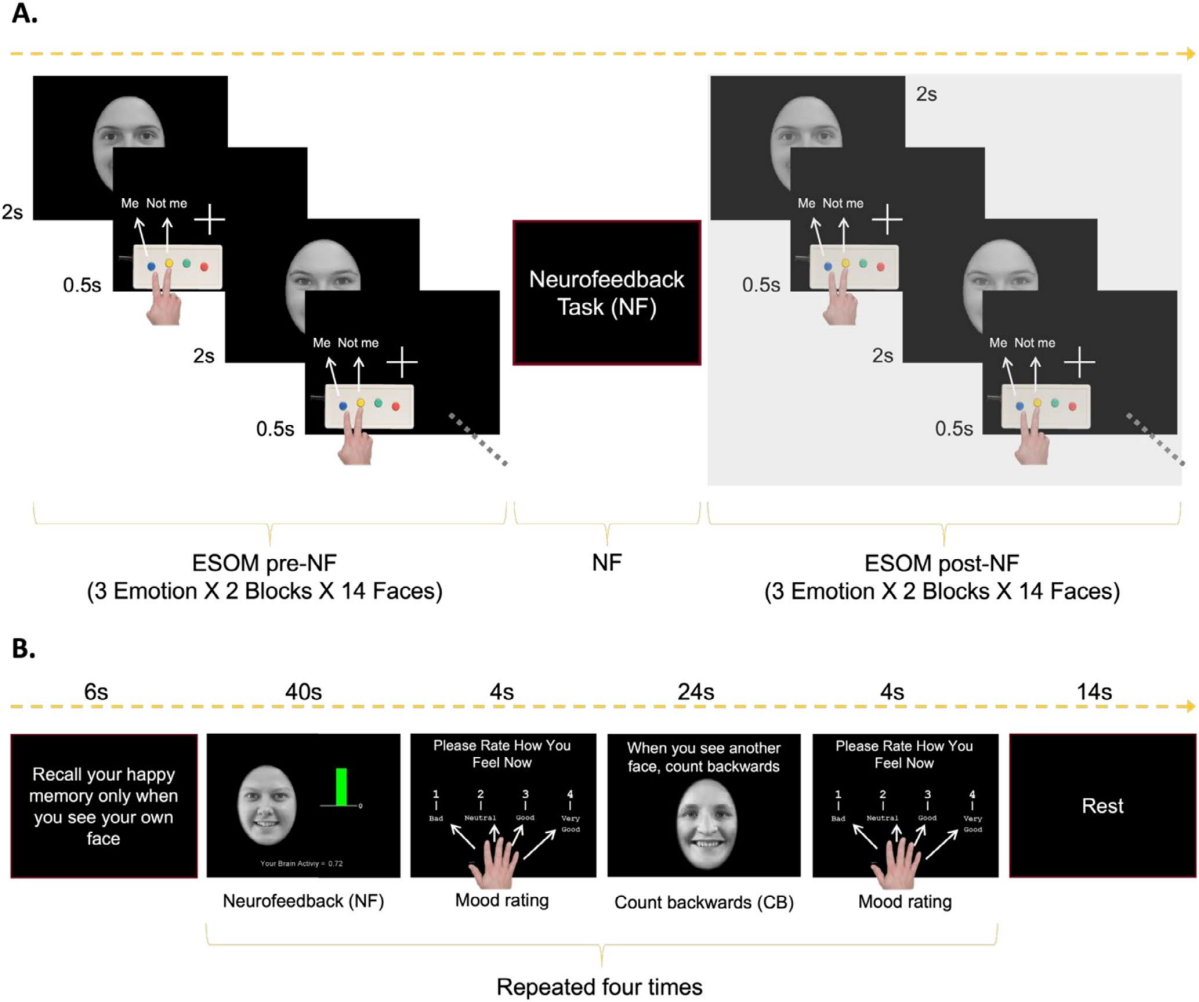
Experimental Design. (A) The overall experiment flow entailed the emotional self‐other morph (ESOM) task pre‐NF, the neurofeedback training (NF), and the ESOM task Post‐NF. Participants identified self or other faces during the Emotional Self‐Other Morph (ESOM) task via button press before and after neurofeedback training. (B) The neurofeedback training was the Emotional Self‐Other Morph Neurofeedback (ESOM‐NF) task. *Neurofeedback*: Participants increased amygdala and hippocampus activity (shown as a changing bar of color: Green = activity > 0, red = activity < 0) paired to images of their own smiling face. Participants recalled positive autobiographical memories to up‐regulate the neural target. *Count backwards*: The control condition entailed counting backwards when seeing an unfamiliar smiling face. *Mood rating*: Participants rated their mood after each block showing faces. For neural network engaged during the training and ratings results see (Quevedo et al. 2019).

To test the effects of real‐time functional magnetic resonance imaging neurofeedback (RT‐fMRI NF) on the circuitry involved in emotion regulation and self‐referential processing, we administered the Emotional‐Self‐Other Morph (ESOM) task to both healthy and depressed adolescents before and after one NF day (Quevedo et al. 2018, 2016). The ESOM task requires participants to recognize pictures of themselves versus unfamiliar faces. The RT‐fMRI NF training paired the self‐happy face with neurofeedback from the AMYHIPP complex, and participants were instructed to increase neural activity by recalling positive autobiographical memories (Figure 1). Given known differences in limbic‐cortical circuitry between depressed and healthy individuals (Carballedo et al. 2011; Quevedo et al. 2018, 2016), we first examined amygdala circuitry changes during the ESOM task after versus before RT‐fMRI NF training paired with the self‐happy face, and second, whether amygdala circuitry during the ESOM task would be associated with performance during the RT‐fMRI NF, which was paired with similar stimuli to regulate amygdala circuitry voluntarily. We also examined how amygdala circuitry during the ESOM task might be associated with rumination or depression improvements.

## 2 Methods

Right‐handed adolescents (*N* = 53, see Table 1 for demographics) were recruited from the Twin Cities community, outpatient, and inpatient units at the University of Minnesota (U of M). Informed consent and assent procedures were administered as approved by the University of Minnesota Institutional Review Board. Exclusion criteria included general MRI exclusions, and meeting diagnostic criteria for psychosis, major medical or neurological disorders, and substance use disorder. However, occasional substance use that did not meet the diagnostic criteria was not excluded. This study was conducted at the Center for Magnetic Resonance Research. Dr. Karina Quevedo, a licensed clinical child psychologist, diagnosed past and current psychiatric disorder (K‐SADS) (Kaufman et al. 1997) and severity of depression (CDRS) (Poznanski et al. 1979) in concordance with a trained research assistant. Medication status, IQ (WASI) (Weschsler 1999), and pubertal status (Petersen et al. 1988) were also ascertained in the first session. Pictures of the participant's face with happy, sad, and neutral expressions were taken following procedures described in Quevedo et al. (2016). Most depressed youth were on stable medication (Table 1) and no participant had to be excluded due to high risks of imminent suicide attempt assessed via the K‐SADS. During the second session, participants identified and wrote 5–6 positive memories and recalled them during NF blocks and completed pretreatment rumination and depression questionnaires (Duration = 1 h). Researchers helped to identify peak positively arousing moments within complex memories. Participants completed the Emotional Self‐Other Morph Neurofeedback task (ESOM‐NF, see Figure 1) during the scanning. Rumination (Treynor et al. 2003) and depression questionnaires (Angold et al. 1995; Messer et al. 1995) were administered before (time 1) and after (time 2) the scanning session (Duration = 1.5 h), with a 4–5 h time gap between completion of baseline questionnaires and follow‐up questionnaires. Participants also reported happiness and ease of recall as reported in Quevedo et al. (2019, 2020). There was no data missing for the participants who attended the NF training.

**TABLE 1:** Demographics and clinical presentation by diagnostic group.

Suicide attempters	Healthy controls	Depressed	Statistics
	*N* = 19	*N* = 34	
	*N* = 0	*N* = 15	
Age at intake: M ± SD	16.26 ± 1.19	16.08 ± 1.27	*F* (1, 51) = 0.26 *p* = 0.612
Age at scanning: M ± SD	16.35 ± 1.23	16.11 ± 1.25	*F* (1, 51) = 0.45 *p* = 0.505
IQ: M ± SD	115.32 ± 9.12	108.35 ± 10.84	*F* (1, 51) = 5.61* *p* = 0.022
Sex			*Χ* ^2^ (1) = 0.31
Male	7 (36.84%)	10 (29.41%)	
Female	12 (63.16%)	24 (70.59%)	
Puberty: M ± SD	4.53 ± 0.65	4.53 ± 0.68	
Ethnicity			*p* = 0.124
White	14 (73.68%)	27 (79.41%)	
African American/Black	0	2 (5.88%)	
American Indian	0	2 (5.88%)	
Asian	3 (15.79%)	0	
Other	2 (10.53%)	3 (8.82%)	
Family structure			*p* = 0.604
Married	15 (78.95%)	22 (64.71%)	
Living with partner	1 (5.26%)	3 (8.82%)	
Separated‐divorced	3 (15.79%)	5 (14.71%)	
Single	0	4 (11.76%)	
Income			*p* = 0.152
≤ 35 K	0	6 (17.65%)	
35–75 K	7 (36.84%)	9 (26.47%)	
≥ 75 K	12 (63.16%)	19 (55.88%)	
Depression before neurofeedback: M ± SD	2.11 ± 2.47	24.26 ± 16.03	*F* (1, 51) = 35.55***
Depression after neurofeedback: M ± SD	2.26 ± 2.47	21.47 ± 16.33	*F* (1, 51) = 25.76***
Rumination before neurofeedback: M ± SD	27.68 ± 6.23	47.18 ± 13.73	*F* (1, 51) = 34.12***
Rumination after neurofeedback: M ± SD	27.89 ± 7.26	44.35 ± 14.35	*F* (1, 51) = 21.75***
Depression severity (CDRS): M ± SD	19.21 ± 3.57	49.85 ± 16.15	*F* (1, 51) = 66.06***
Diagnosis (K‐SADS‐PL)			
Major depressive disorder (MDD)	0	14	
MDD with psychotic features	0	1	
Dysthymia	0	4	
Melancholic depression	0	1	
Depressive disorder‐NOS	0	15	
Eating disorders	0	2	
Anxiety disorders	0	22	
PTSD	0	6	
Disruptive behavior disorders	0	6	
Substance use experimentation, but no substance use disorder	0	2	
Medication			
Antidepressants	0	26	
Antipsychotics	0	2	
Mood stabilizers	0	0	
Anxiolytics	0	10	

*Note:* Fisher's Exact Test was applied due to small cell counts for ethnicity, family structure, and income.

Abbreviations: M, mean; SD, standard deviation.

*p < 0.05, **p < 0.01, ***p < 0.001.

### 2.1 Tasks

#### 2.1.1 Emotional Self‐Other Morph Query (ESOM‐Q) Task

An initial rest (Duration = 30 s) was followed by an instruction (Duration = 6 s.). A shorter version (total duration = 360 s.) of the ESOM task (Quevedo et al. 2018, 2016) was presented before (ESOM_Pre‐NF) and after (ESOM_Post‐NF) the neurofeedback (ESOM‐NF) task. Participants identified 168 faces with high or low percentages of self‐features across three emotions (Happy, Neutral, or Sad) via button press to indicate their own or an unfamiliar face. The stimuli were blocked by self or other faces, which were presented in the same order during ESOM_Pre‐NF and ESOM_Post‐NF. Each block lasted 35 s followed by a rest period (duration = 18 s). Instructions were presented for 3 s before each condition. Within each self or other‐face block, there were 2 instances of the opposite face condition to diminish response sets and maintain attention (e.g., during the self‐face block, there were 2 other faces). Stimuli were presented and reaction time (RT) and accuracy were collected using PsychoPy software (Peirce 2007) (Figure 1).

#### 2.1.2 Emotion Self‐Other Morph Neurofeedback (ESOM_NF)

This task (Total duration = 354 s.) consisted of four blocks of neurofeedback (40 s) and count‐backward (24 s.; control condition) followed by a rating on their affect (1 = bad to 4 = good) after each trial. The first block started with the participant seeing their own happy face and was asked to increase AMYHIPP activity displayed in a colored bar shifting up or down continuously depending on values provided by MURFI software (Hinds et al. 2011), with green indicating the activity higher than the baseline and red indicating the activity lower than the baseline (Figure 1). As a result, participants completed a single neurofeedback session consisting of four repetitions (i.e., four blocks). To modulate AMYHIPP activity, participants recalled happy autobiographical memories and were verbally instructed both before and during the task to elicit the positive feelings: “try to feel what you felt then”. The control condition was paired with an unfamiliar teen happy face and participants counted numbers backwards from 100 in this no‐feedback condition. It was expected that this would engage areas supportive of both working memory (Woodward et al. 2006) and face processing (Sabatinelli et al. 2011) but not those uniquely elicited by recalling positive autobiographical memories and self‐face recognition during neurofeedback (Alarcon et al. 2019; Quevedo et al. 2018, 2016). The order of the neurofeedback and count‐backward trials alternated throughout the training (Figure 1). Three rests occurred at the start (30 s.), in the middle (Time point = 80 s., duration = 2 s.) and the end of the task (Time point = 342 s., duration = 12 s.) comprising an implicit baseline condition. Participants saw instructions (6 s) after the first and second rests. Rest/baseline and count‐backward conditions have served as contrasts to test neuromodulation's effects on the region of interest (ROI) during neurofeedback. This contrast allowed us to test neural representations supporting the recognition of self‐other face and how the network is affected by the use of those stimuli to prompt voluntary modulation (self‐face recognition) versus a different mental activity (other's face recognition).

### 2.2 Magnetic Resonance Imaging (MRI) Data Acquisition and Processing

Neuroimaging data were collected using a 3.0 Tesla Siemens Prisma MRI scanner with the 32‐channel receive‐only head coil. Structural 3D axial MPRAGE images were acquired for each participant (TR/TE: 2100 ms/3.65 ms; TI: 1100; Flip Angle: 7°; Field of View: 256 × 256 mm; Slice Thickness: 1 mm; Matrix: 256 × 256; 224 continuous slices) using GRAPPA 2 acceleration. Mean BOLD images were then acquired with a slice‐accelerated gradient echo EPI sequence during 6.08 min for the ESOM_Pre and Post tasks (2.4 mm^3^ voxels, covering 60 oblique axial slices; TR/TE = 1510/32.4 ms; FOV = 216 × 216 mm; matrix 90 × 90; Flip Angle: 65°; multi‐band acceleration factor: 3).

SPM12 was used for fMRI preprocessing and statistical analyses. Preprocessing the EPI time series included: (1) rigid body realignment for head motion correction, (2) slice timing correction, (3) rigid body co‐registration of EPI with high‐resolution anatomical data, (4) spatial normalization to the Montreal Neurological Institute (MNI) anatomical space using unified segmentation, and (5) spatial smoothing (6 mm full width at half maximum). Head motion outliers in EPI time series were identified using the Artifact Detection Tools with a scan‐to‐scan movement threshold of 0.5 mm and a scan‐to‐scan global signal change of 3 SD (www.nitrc.org/projects/artifact_detect/). For each subject, BOLD‐contrast signal variance was modeled with a set of regressors using a general linear model. The total signal variance was decomposed into a task component, with intertrial intervals as implicit baselines. Each task regressor was constructed by generating condition duration vectors and then convolving them with a canonical hemodynamic response function, allowing parameter estimates proportional to task‐related neural activity per second. The full model for each subject comprised: (1) the condition regressors, (2) regressors modeling movement‐related signal modulation, (3) outlier time points, (4) the mean signal for the session, and (5) a discrete cosine transforms basis set that modeled the low‐frequency, presumably artifactual, signal modulations at frequencies lower than 0.008 Hz. Parameter estimates were calculated using the restricted maximum likelihood algorithm.

### 2.3 Online Analyses

MURFI software (Hinds et al. 2011) generated and sent AMYHIPP activity values during the feedback condition displayed with PsychoPy (Peirce 2007) during the Emotion Self‐Other Morph Neurofeedback task using subject‐specific anatomical masks of the bilateral amygdala and hippocampus. The bar representing AMYHIPP activity values was updated continuously as each new functional magnetic resonance imaging (fMRI) volume was acquired. Online subject head motion compensation was accomplished using the Siemens PACE/MoCo system (Thesen et al. 2000). Feedback automatically stopped if movement exceeded 4–3 mm repeatedly (which occurred in just one participant), but participants could re‐initiate the Emotion Self‐Other Morph Neurofeedback task. Data were not corrected for other physiological signals such as respiration‐related variables. Region of interest (ROI) was localized anatomically during the multiband echo‐planar imaging (EPI) series (target functional reference acquisition) for each individual and mapped to the individual's T1 structural brain data. Data were collected using a 3.0 Tesla Siemens Prisma MRI scanner with the 32 Channel receive‐only head coil. Structural 3D axial MPRAGE images were acquired for each participant (TR/TE = 2100 ms/3.65 ms; TI = 1100; Flip Angle = 7°; Field of View = 256 × 256 mm; Slice‐Thickness = 1 mm; Matrix = 256 × 256; 224 continuous slices), GRAPPA 2. Mean blood oxygen level dependent (BOLD) images were then acquired with a slice‐accelerated gradient echo‐planar imaging sequence during 6.08 min for the Pre‐ and Post‐ Emotion Self‐Other Morph tasks and 6.02 min for the Emotion Self‐Other Morph Neurofeedback task (60 oblique axial slices; TR/TE = 1510/32.4 ms; Field of View = 216 × 216 mm; Matrix = 90 × 90; Flip Angle = 65°; Voxel size = 2.4 mm^3^; multi‐band acceleration factor 3).

### 2.4 Off‐Line Analyses

Functional connectivity analyses were conducted using SPM 12. The rest of the analyses were conducted using IBM SPSS Statistics (Version 29). Graphs are generated using R Studio (version 4.4.1). Seed regions for connectivity analyses were 8 mm spheres placed in the left (roughly centered at, left: −30, −2, −28 and right: 32, 0, −30) amygdala. The left and right amygdala regions of interest masks were defined by the Pick Atlas toolbox (Maldjian et al. 2003). The time series of peak amygdala activity was extracted for each participant for 8 mm spheres centered in those individual highest activity coordinates within the amygdala regions of interest. This resulted in slightly different peak coordinates per participant that reflected the highest amygdala activity for each individual youth within the boundaries of the amygdala. Amygdala activity peak coordinates did not statistically differ by group, *F* (3, 104) = 1.79, *p* = 0.154, or gender, *F* (1, 104) = 3.81, *p* = 0.054. In addition, we performed intraclass correlation coefficient (ICC) analyses with time series, using left and right hippocampus and left and right amygdala derived from Pick Atlas software (Maldjian et al. 2003), to investigate the degree of coordination between the amygdala and the hippocampus during all the significant effects reported in the manuscript (Group × Time, Group × Hemisphere, and Time × Hemisphere) for ESOM pre‐ and post‐NF activities. These are covered in the discussion section.

#### 2.4.1 Functional Connectivity (FC) Analyses

FC was estimated via psychophysiological interactions (PPI) analysis (Data S1, Figure S1) to explain activity changes in the target region based on an interaction between amygdala activity and self‐other face recognition, a psychological variable, during the ESOM task (Friston et al. 1997). Amygdala and hippocampus FC between groups during the ESOM_Pre‐NF and Post‐NF tasks were examined. Signal time course in left or right amygdala was obtained for each participant and task condition to construct the PPI variable. The contrast of interest for analysis was differences between self and other faces recognition (self‐other faces). The resulting first‐level PPI activation maps for ESOM_Pre‐NF and Post‐NF were subjected to a 2nd level general linear model (GLM) with one between‐group factor, diagnosis (Depressed vs. Controls), and two within‐group factors, time (2 levels: Pre and Post) and amygdala hemisphere (2 levels: Left and Right). Variables that differed by groups—IQ and medication—were added as covariates. Significantly engaged brain areas represented areas with high or low connectivity with the amygdala or hippocampus. The above GLM was further explored in follow‐up one‐way ANOVAs masking for significant areas of connectivity yielded by the “Diagnosis by Time by Amygdala Hemisphere” GLM to confirm the direction of results (i.e., increase or decrease in connectivity over time by group). Clusters and coordinates with statistically significant peak BOLD signals (*p* < 0.001) are reported. Similar analyses for the hippocampus and the hippocampus‐amygdala complex were run but showed no significant connectivity patterns and thus are not further discussed. Only amygdala connectivity analysis yielded significant results and is reported.

Smoothness estimates from 3dClustSim (12.60 12.25 13.21) in AFNI (v. 18.2.06) were used to correct for multiple comparisons and determine whole brain, voxel‐wise and cluster‐extent thresholds (Cox 1996). For the principal GLM including ESOM_Pre‐NF and Post‐NF images a threshold of *α* = 0.01, *p* < 0.001 yielded a minimum threshold of 162 voxels to reach significance. Smoothness estimates entered into 3dClustSim were averages of subject‐level spatial autocorrelation function (acf) parameters based on individual subjects' residuals from group‐level models, as calculated by 3dFWHMx. Pearson correlation values significant at the 0.01 or 0.05 level were obtained and reported as significant correlates for changes in rumination or depression and brain areas identified in the group (depressed, controls) by hemisphere (left, right amygdala) interaction. Significant estimates of FC were extracted using the SPM12 “eigenvariate” function to produce plots depicting the direction of effects (i.e., positive or negative relationships between connectivity and rumination or depression).

#### 2.4.2 AMYHIPP Activity During NF Analyses

Region of interest analyses (ROI) in prior work confirmed AMYHIPP higher activation for NF compared to count‐backward conditions (Quevedo et al. 2019, 2020). Similar to the prior work, mixed effect linear models (MLMs) were fitted with the mean BOLD activity within subjects for the 8 blocks of ESOM‐NF as the outcome variable. Individual subjects' AMYHIPP values during ESOM‐NF were predicted by amygdala connectivity estimates during self‐versus‐other face recognition after versus before NF training along with other predictors using a hierarchical linear model (HLM).

ROI analyses used individual mean signals across all conditions in comparison to the implicit baseline, after movement correction. Individual intercepts (β0i) were modeled as random effects and mean intercept (γ00), longitudinal trends (linear, quadratic, cubic, and quartic slopes) and predictors of interest as fixed effects. Linear change over time and inflection points during ESOM‐NF were modeled with linear (γ01), quadratic (γ02), cubic (γ03), and quartic (γ04) polynomials. The initial model had 22 predictors which were removed one at a time starting with the least significant predictor of AMYHIPP activity during the NF task.

An identity covariance structure for the random effects fitted best for all the time series. We used a two‐stage strategy for model selection. In the first stage, we used the lowest Akaike information criterion (AIC) statistic to select anchor models. The AIC, however, is known to suggest overly complex models (Kass and Raftery 1995). Thus, in a second stage we compared nested models (larger models compared to nested smaller models) via a likelihood ratio test to refine the anchor model selected by the AIC. Final models included key predictors, including interactions, but could exclude main effects if simpler models with just the interaction terms better fitted the data (Singer and Willett 2003). Simple slopes tests, keeping all other predictors at mean values, confirmed the direction of interactions (Table 2).

**TABLE 2:** Amygdala connectivity during self‐other face recognition tasks in ESOM_Pre and ESOM_Post Neurofeedback (NF) training.

Whole‐brain results	Voxels	H	MNI coordinates			*F*	*p*	$\eta_{p}^{2}$
			*x*	*y*	*z*			
**Group by time interaction during self‐other face recognition**								
Superior and Middle Temporal Gyrus, BA22, 41	256	R	50	−34	12	21.74	< 0.001	0.25
Superior and Middle Frontal Gyrus, BA10	217	R	28	42	22	18.13	< 0.001	0.23
Anterior Cingulate Cortex, BA24, 32	181	R & L	10	20	30	14.92	< 0.001	0.23
**Group by amygdala hemisphere interaction during self‐other face recognition**								
Cuneus, BA17	162	R	14	−84	−02	13.84	< 0.001	0.17
**Time by amygdala hemisphere Interaction during self‐other face recognition**								
Middle Temporal Gyrus, BA 21	167	R	56	−24	−06	19.68	< 0.001	0.26
Superior and Middle Temporal Gyrus, BA22, 40	258	R	56	−38	04	15.98	< 0.001	0.22
Middle Frontal Gyrus, BA9	177	R	46	26	38	15.45	< 0.001	0.22

*Note:* Only the significant results are included.

Abbreviations: $\eta_{p}^{2}$, partial eta‐squared; H, hemisphere; L, left; R, right.

**FIGURE 2:**
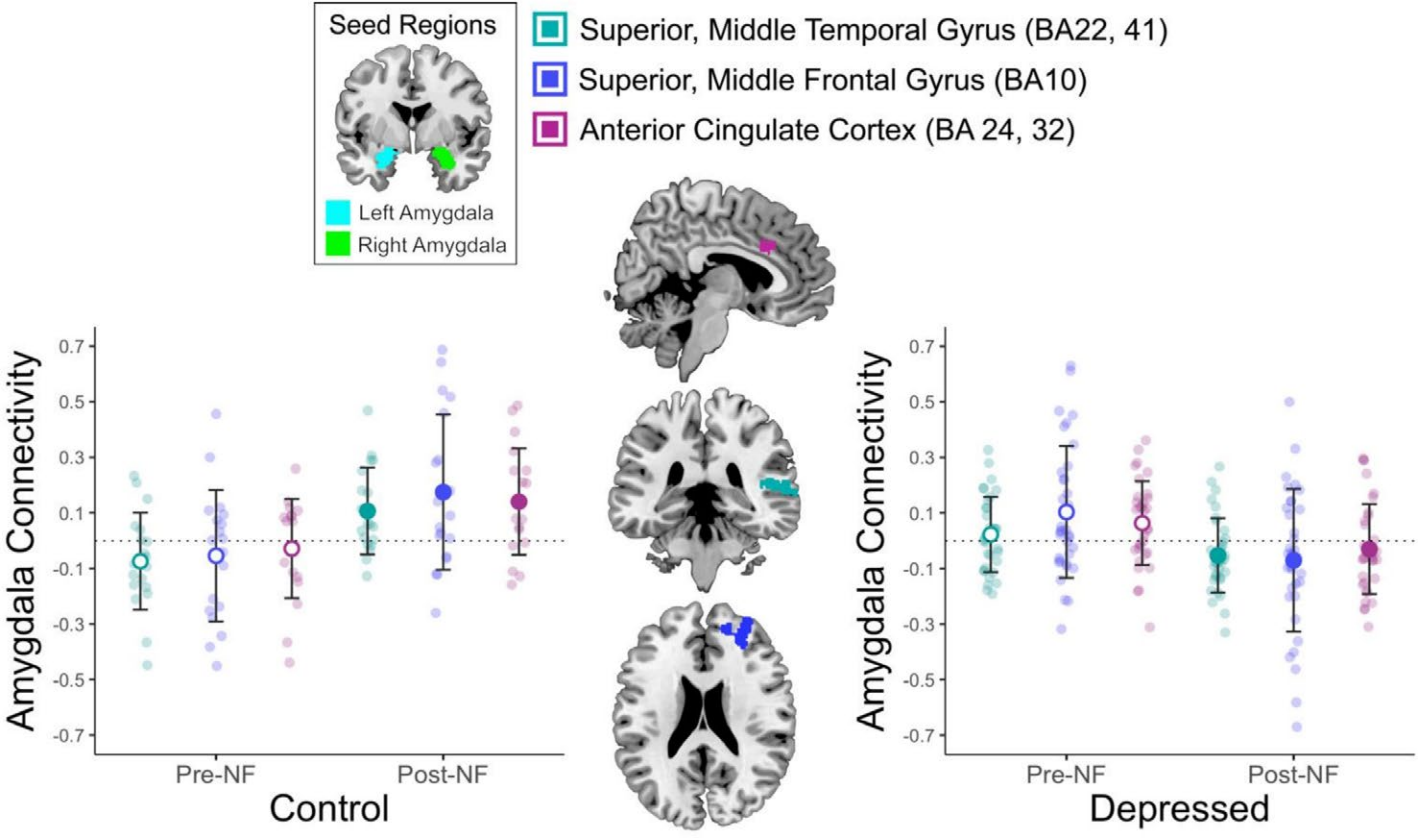
Group by Time Interaction in Amygdala Connectivity. Depressed youth showed higher bilateral amygdala connectivity to superior middle temporal gyrus, superior middle frontal and ACC, during self‐other face recognition before neurofeedback training; but they showed lower connectivity after neurofeedback (NF) during self‐other face recognition. By contrast healthy controls showed lower bilateral amygdala connectivity to superior middle temporal gyrus, superior middle frontal gyrus, and ACC connectivity during self‐other face recognition before NF, but higher connectivity after NF. Error bars depict mean ±1 standard deviation.

**FIGURE 3:**
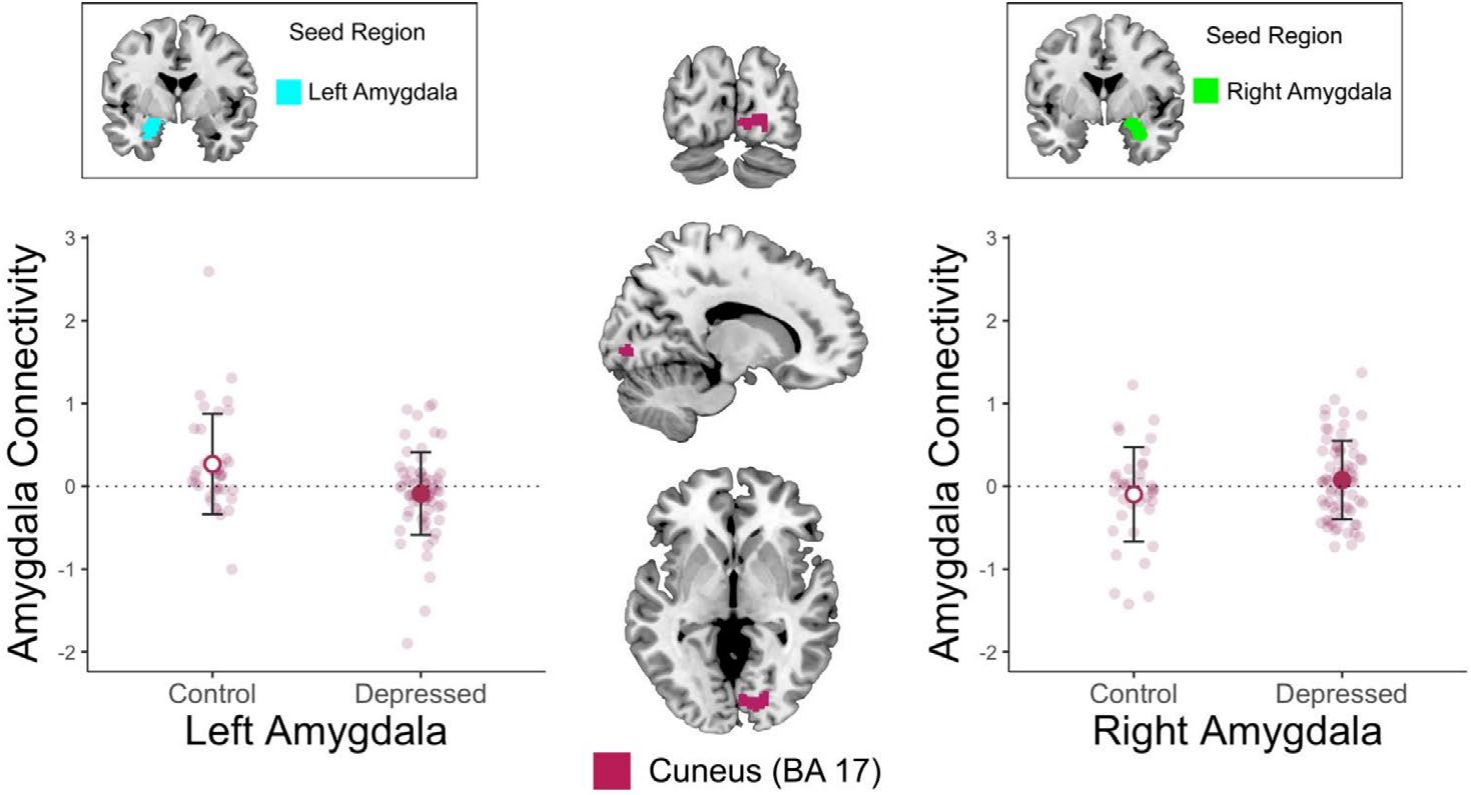
Group by Amygdala Hemisphere Interaction in Connectivity during Self‐Other Face Recognition. Control participants show high left amygdala‐cuneus connectivity while depressed youth show close to 0 left amygdala‐cuneus connectivity; by contrast depressed youth show higher right amygdala‐cuneus connectivity while control youth showed lower right amygdala‐cuneus connectivity. Error bars depict mean ±1 standard deviation.

Significant functional connectivity (FC) estimates for the ESOM_Post and Pre GLM analyses (Table 2) were synthesized for all participants. For the *Group by Time Interaction* (Figure 2), each estimate of amygdala functional connectivity was averaged across the amygdala hemisphere and subtracted to reflect time differences (ESOM_Post—ESOM_Pre) in amygdala connectivity. For the *Group by Amygdala Hemisphere Interaction*, FC estimates (Figure 3) were synthesized by averaging FC estimates across ESOM_Pre and ESOM_Post and subtracting the left from the right amygdala FC estimates. For the *Time by Amygdala Hemisphere Interaction*, FC estimates were synthesized by subtracting FC estimates of the left from the right amygdala for each ESOM_Post and ESOM_Pre neurofeedback task, and then subtracting the resulting FC estimates of ESOM_Pre from ESOM_Post. For example (left amygdala—right amygdala FC) ESOM_Post—(left amygdala—right amygdala FC) ESOM_Pre. These three summary variables were entered as predictors in the HLM to predict AMYHIPP activity time series during the ESOM‐NF training, along with other predictors of interest such as group, gender, IQ, etc.

## 3 Results

### 3.1 Group by Time Interaction in Amygdala Connectivity

Superior middle frontal and temporal gyri (BA 22, 41, 10) and ACC (BA 24, 32) to bilateral amygdala connectivity varied by time for the diagnostic groups during self‐other face recognition in the ESOM task. Before NF, depressed youth showed *higher* bilateral amygdala to ACC, superior temporal gyri, and frontal gyri connectivity during self‐other face recognition compared to control youth, who instead showed *lower* bilateral amygdala connectivity to those areas before NF (Figure 2, Table 2). After NF, depressed youth showed lower bilateral amygdala connectivity to ACC, superior temporal, and frontal gyri compared to control youth during self‐other face recognition (Figure 2).

### 3.2 Group by Amygdala Hemisphere Interaction in Amygdala Connectivity

Left and right amygdala to cuneus connectivity differed between groups during the self‐other face recognition, ESOM task (Figure 3, Table 2). Specifically, depressed youth showed significantly lower left amygdala to cuneus (BA 17) connectivity than controls, but they showed a significantly higher right amygdala to cuneus connectivity compared to control youth (Figure 3) during self‐other face recognition regardless of time. In other words, depressed youth had higher connectivity between the right amygdala to cuneus when engaging in self‐other face recognition, irrespective of time (Table 2). These results show differences in explicit versus implicit emotion processing supported respectively by the left versus right amygdala circuitry that are evident in depressed versus healthy control youth.

### 3.3 Rumination and Depression Improvement and Amygdala Circuits

The correlation between depression and rumination improvement and amygdala‐cuneus connectivity during the self‐other face recognition neurofeedback was separately examined for each amygdala hemisphere in depressed youth (*N* = 34).

**FIGURE 4:**
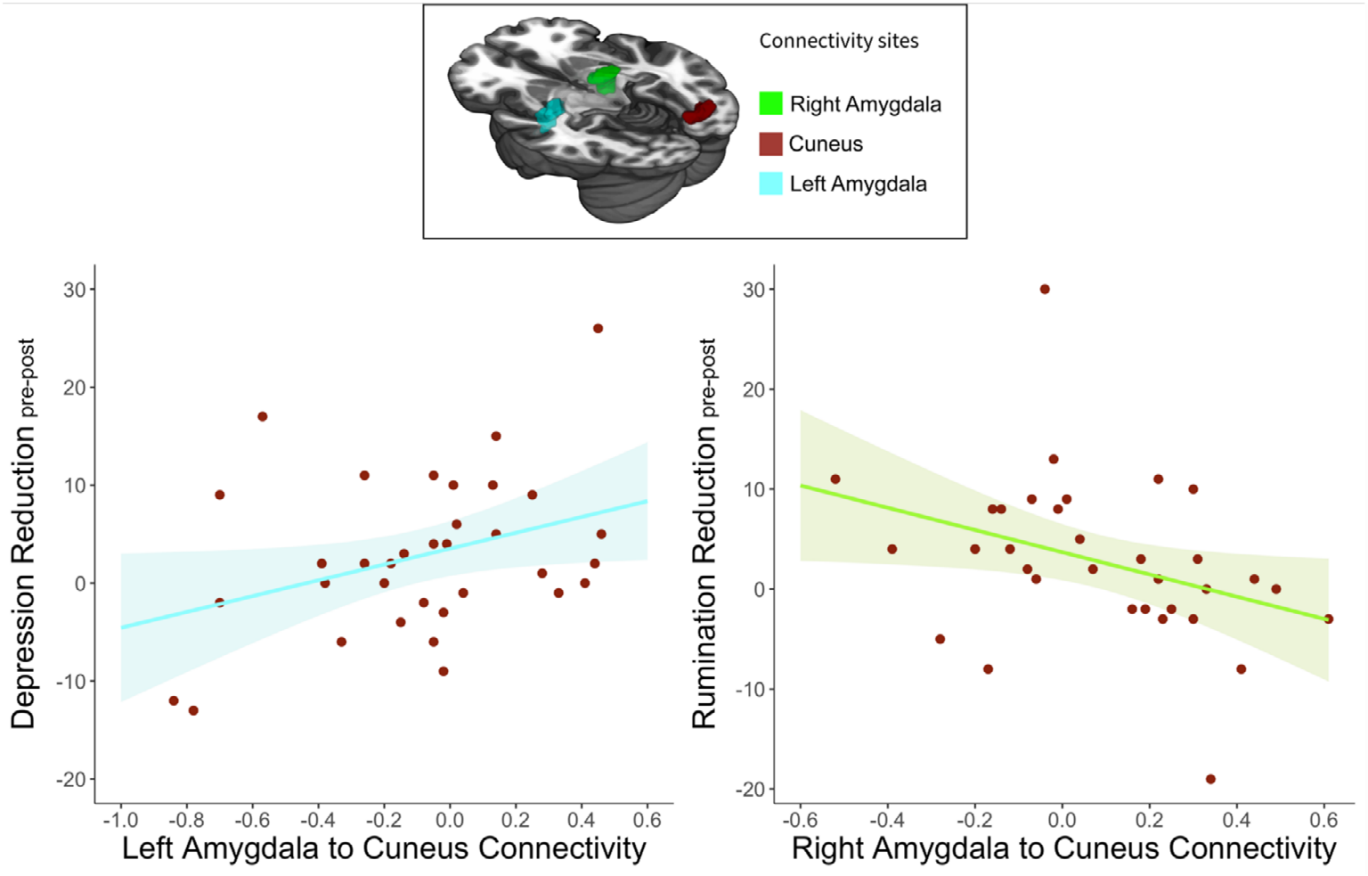
Correlation between Amygdala and Cuneus Connectivity with Depression and Rumination Improvement among Depressed Youth. Stronger connectivity between cuneus and left amygdala, during self or other face recognition, was related to higher depressive symptom reduction and weaker connectivity with right amygdala was related to higher rumination reduction. Rumination reduction = rumination pre neurofeedback minus rumination post neurofeedback. Shaded areas show 95% confidence intervals.

Among depressed youth, those with *lower* right amygdala to cuneus connectivity, during the self‐other face recognition, ESOM task (Figure 3), showed a greater reduction in rumination, *r* = −0.36, *p* = 0.037 (Figure 4). The depressed youth with *higher* left amygdala to cuneus connectivity during the ESOM task, showed a greater reduction in depression, *r* = 0.35, *p* = 0.042 (Figure 4). There was no relationship between the right amygdala to cuneus connectivity during the task with depression change, *r* = −0.24, *p* = 0.167, and the left amygdala to cuneus connectivity during the task with the rumination change, *r* = 0.24, *p* = 0.176. In summary high left amygdala‐cuneus and low right amygdala‐cuneus activity during self‐other face recognition, were linked to rumination improvement in depressed youth. This finding is important because as Figure 3, shows depressed youth showed lower left amygdala and higher right amygdala to cuneus connectivity compared to controls, suggesting that this circuit is a marker of individual differences in emotion dysregulation improvements among depressed youth.

Correlations between symptom improvements and amygdala‐cuneus connectivity were conducted by hemisphere and group as well (Data S2, Figure S2). These analyses showed that as the connectivity between the left amygdala and cuneus strengthens after the NF task, rumination symptoms were reduced for the depressed group only, *r* = 0.37, *p* = 0.032. This was not observed in the control group. The supplements also report that rumination and depression scores were strongly positively related both before and after the NF (Data S3, Figure S3).

### 3.4 Amygdala Connectivity During Self‐Other Face Recognition and AMYHIPP Activity Changes During Neurofeedback Paired to the Self‐Face

**FIGURE 5:**
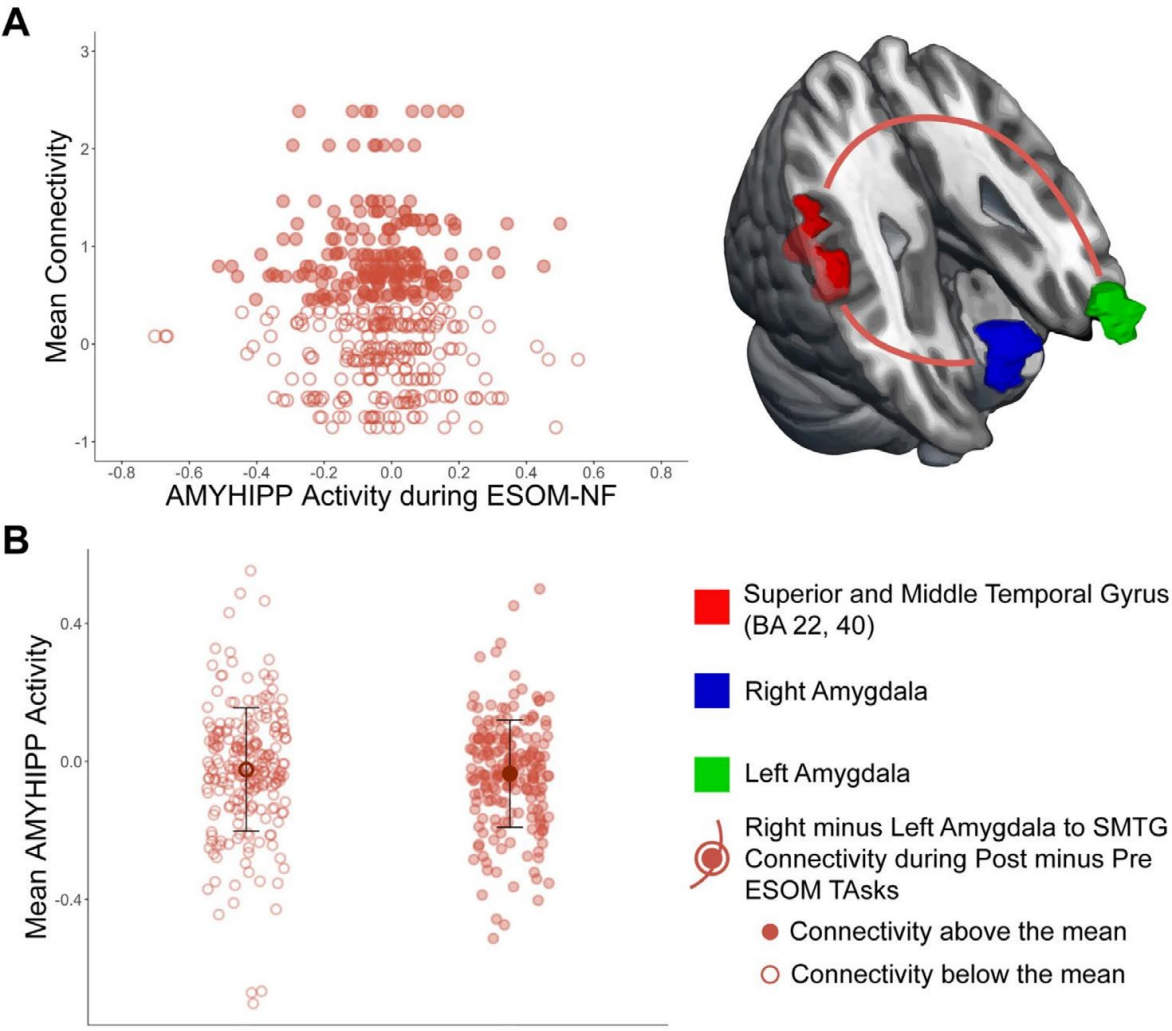
Amygdala and Hippocampus Modulation was Predicted by Right minus Left Amygdala to Superior Temporal Gyrus Connectivity during Post versus Pre ESOM tasks. (A) Association between STG to right minus left amygdala connectivity after minus before neurofeedback (*y* axis) and mean amygdala and hippocampus (AMYHIPP) activity during neurofeedback (*x* axis). Lower right versus left amygdala connectivity to STG after versus before neurofeedback training was associated with higher amygdala and hippocampus activity during neurofeedback. (B) Mean amygdala and hippocampus activity (*y* axis) during neurofeedback split by right minus left amygdala‐STG connectivity below and above the mean. *Below the mean* right versus left amygdala‐STG connectivity after versus before neurofeedback training is linked to higher AMYHIPP activity; while *above the mean* right versus left amygdala‐STG connectivity after versus before neurofeedback is linked to lower AMYHIPP activity. Error bars depict mean ±1 standard deviation.

Lower superior temporal gyrus (STG) and middle temporal gyrus (MTG), to right amygdala connectivity versus left amygdala connectivity during self‐other face recognition after versus before neurofeedback training (Data S4, Figure S4) were associated with higher AMYHIPP activity during the neurofeedback training (Table 3, Figure 5).

**TABLE 3:** Predictors of amygdala and hippocampus activity during the ESOM‐NF task.

Effect	Estimate *ϒ*	SE *ϒ*	df	*t*	95% CI	*p*	*d*
Intercept	0.09	0.03	114	2.95	[0.03, 0.15]	0.004	
Linear Slope	−0.15	0.04	371	−3.30	[−0.24, −0.06]	0.001	0.17
Quadratic Slope	0.11	0.03	371	3.87	[0.05, 0.17]	< 0.001	0.20
Cubic Slope	−0.03	0.006	371	−4.26	[−0.04, −0.01]	< 0.001	0.22
Quartic Slope	0.002	0.0004	371	4.46	[0.001, 0.003]	< 0.001	0.23
ESOM‐NF Condition							
NF + Happy Self‐Face	0.07	0.01	371	−4.54	[−0.10, −0.04]	< 0.001	0.23
CB + Happy Other‐Face (ref)							
Medication							
Absence	−0.09	0.04	53	−2.27	[−0.16, −0.01]	0.028	0.30
Presence (ref)							
Gender							
Males	0.05	0.02	53	−2.04	[−0.10, −0.0008]	0.047	0.28
Females (ref)							
Group							
Control	0.13	0.04	53	3.17	[0.05, 0.21]	0.003	0.43
Depressed (ref)							
SMTG to AMY (Right vs. Left) connectivity during ESOM_Post‐NF versus ESOM_Pre‐NF	−0.04	0.02	53	−2.43	[0.008, 0.08]	0.018	0.33
MFG to AMY (Right vs. Left) connectivity during ESOM_Post‐NF versus ESOM_Pre‐NF	0.02	0.01	53	1.82	[−0.05, 0.002]	0.074	—

*Note:* Reference groups or conditions are marked with “(ref).”

Abbreviations: CB, count‐backward; MFG, middle frontal gyrus; SMTG, superior and middle temporal gyrus.

**FIGURE 6:**
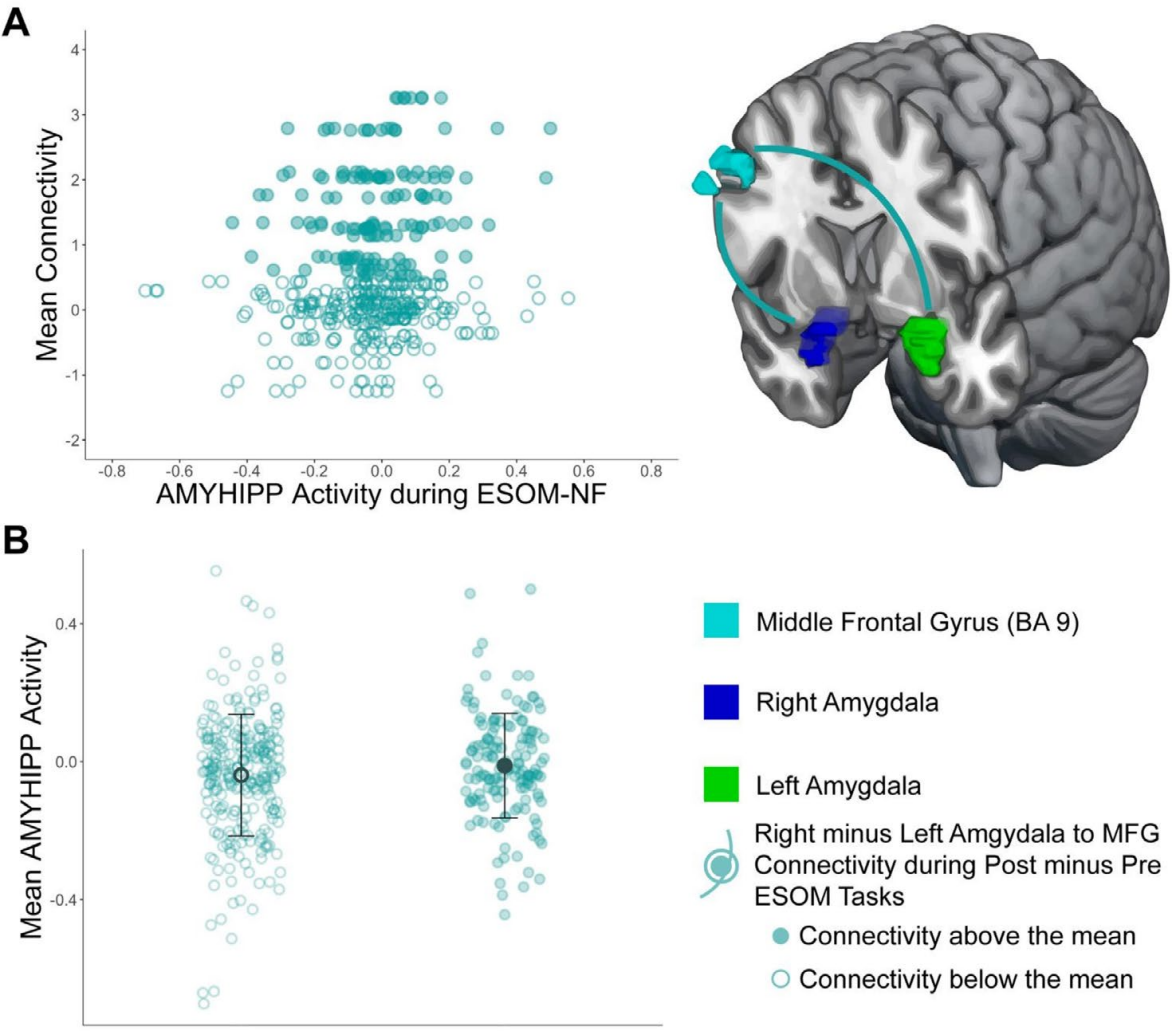
Amygdala and Hippocampus Modulation Predicted by Right minus Left Amygdala to Middle Frontal Gyrus Connectivity during Post versus Pre ESOM tasks. (A) Association between mean connectivity of MFG to right minus left amygdala during ESOM Post‐NF minus ESOM Pre‐NF tasks (*y* axis) and mean amygdala and hippocampus activity during NF training (*x* axis). Higher right versus left amygdala connectivity to MFG after versus before neurofeedback training was associated with higher amygdala and hippocampus activity during neurofeedback. (B) Mean amygdala and hippocampus activity during NF (*y* axis) split by right minus left amygdala‐MFG connectivity below or above the mean. *Above the mean* right versus left amygdala‐MFG connectivity after versus before neurofeedback training is linked to higher AMYHIPP activity; while *below the mean* right versus left amygdala‐MFG connectivity after versus before neurofeedback is linked to lower AMYHIPP activity. Error bars depict mean ±1 standard deviation.

In addition, higher middle frontal gyrus, MFG (BA 9) to right amygdala connectivity versus left amygdala connectivity during self‐other face recognition after versus before neurofeedback training (Data S5, Figure S4) showed a trend to be linked to higher AMYHIPP activity during the neurofeedback training (Table 3, Figure 6).

We also replicated prior findings (Quevedo et al. 2019, 2020) including higher AMYHIPP activity levels during NF compared to the count‐backward condition (i.e., successful neurofeedback), higher AMYHIPP activity for control compared to depressed youth, and for males compared to females (Table 3). The linear to quadratic slopes showed the trend of higher values for NF, with a steeper decline during the count‐backward condition.

## 4 Discussion

We have previously shown that neurofeedback training examined in this manuscript is effective at reducing depressive and rumination symptoms, with an average 30% decrease in the mood and feelings questionnaire (Quevedo et al. 2020). Specifically, Quevedo et al. showed that high right amygdala connectivity to CMS was associated with lower rumination, whereas low left amygdala connectivity was linked to lower depression (Quevedo et al. 2020). The current analysis focused on the amygdala connectivity involved in self‐referential processing before versus after an amygdala‐targeted neurofeedback training procedure known as the ESOM‐NF task which paired amygdala and hippocampus neurofeedback to an image of the smiling participant's face and entailed up‐regulating the target's activity levels by recalling positive autobiographical memories.

Two important results were observed. First, there were significant differences in bilateral amygdala connectivity with CMS, specifically the ACC (BA 24, 32). Between the diagnostic groups. Compared to controls, depressed youth showed higher amygdala connectivity during self‐referential processing *before* NF and showed lower amygdala connectivity *after* NF with CMS including ACC as well as superior middle temporal and superior middle frontal gyri. By contrast, control participants showed the opposite pattern of results (Figure 2). Second, depressed individuals showed higher right amygdala to cuneus connectivity during self‐other face recognition, while healthy control youth showed higher left amygdala to cuneus connectivity (Figure 3). It should be noted that the participants were exposed to a single session of neurofeedback entailing four neurofeedback training runs, so our results could improve with increased exposure and training sessions over time.

### 4.1 Amygdala‐Cortical Connectivity During Self‐Other Face Recognition Before vs. After Neurofeedback

Previous studies have demonstrated that depressed adults exhibit high connectivity between the middle frontal gyrus and dorsal anterior cingulate cortex (dACC) during self‐referential processing (Lemogne et al. 2009). Similarly, depressed patients show higher connectivity between the visual cortex and the amygdala during emotionally challenging tasks, such as evaluating negative and positive pictures, indicating biased valence processing (Tozzi et al. 2017). There are several reports of amygdala‐cortico‐medial structures (CMS) *hyper‐connectivity* during emotionally challenging or self‐relevant tasks (Fowler et al. 2017; Murphy et al. 2016). Hyper‐connectivity, in turn, has been linked to high negative affect, and connectivity changes predict adolescent depression's onset (Davey et al. 2015). Consistent with previous research, our findings demonstrate high bilateral amygdala‐CMS connectivity in depressed youth before neurofeedback, which decreased after the neurofeedback training. These findings align with prior research that has demonstrated decreased activity in the same three clusters (dACC, SFG and MFG) in individuals with MDD (Canli et al. 2004; Drevets et al. 2008, 1997; Fitzgerald et al. 2008; Taylor and Liberzon 2007). This reduced activity has been interpreted as reduced “top‐down” cognitive and executive control. The higher amygdala input to CMS reported here prior to NF and low CMS activity in cited literature suggest a preponderance of limbic emotional inputs to the detriment of regulatory/executive processes in depressed patients.

The presence of high cortical connectivity with the bilateral amygdala during the initial self‐other face recognition task in depressed youth, prior to neurofeedback, which is subsequently reversed after neurofeedback, might indicate a dominant influence of emotions over executive control processes during self‐processing in depressed compared to healthy youth. Given that connections between the amygdala and cortical regions likely influence emotion‐guided behaviors (Price and Glad 2003), and that MDD is associated with an overactive amygdala (Hamilton and Gotlib 2008), we speculate that neurofeedback might have shifted emotional contributions borne by the amygdala to higher‐order cognitive and emotional control mediated by the PFC and reversed its overactive role in social and emotional cognition. However, in the absence of a placebo group, alternative explanations, such as habituation to self‐recognition or amygdala habituation cannot be ruled out. For example, any source of habituation may lead to reduced emotional reactivity or increased task performance efficiency. Furthermore, our interpretations regarding connectivity are speculative and incorporating measures of executive and cognitive control pre and post intervention into future studies would allow us to directly test this proposed mechanism.

Regarding the healthy controls, previous research shows that increased amygdala‐mPFC connectivity, which reflects amygdala inhibition by the mPFC, is correlated with better emotion regulation during voluntary regulation of negative emotion via cognitive reappraisal (Banks et al. 2007). In the context of our study, the lower amygdala‐cortical connectivity observed in healthy controls before versus after neurofeedback might indicate a reduced need for negative emotion regulation during self‐recognition in healthy youth. Subsequently, the increase in amygdala‐CMS connectivity after neurofeedback may reflect either higher emotional saliency or enhanced executive control after the neurofeedback procedure (Figure 2). In summary, amygdala‐CMS connectivity patterns for controls versus depressed youth—with the latter exhibiting less amygdala‐CMS connectivity after NF—might indicate predominant limbic inputs that decrease due to either habituation or to NF training in depressed youth. It is important to note that the precise functional implications of this increase in amygdala‐CMS connectivity in controls during self‐recognition cannot be fully elucidated in the present study. Future research is needed to validate these speculative explanatory frameworks, as the current analyses do not provide information on the directionality of connectivity, and we did not have concurrent measures of emotional saliency or executive/cognitive control. It is worth mentioning that contrasting findings have been reported in studies involving depressed adults, where initial amygdala connectivity with cortical areas is low and NF increases connectivity (Li et al. 2016; Wang et al. 2019; Yuan et al. 2014). It is important to consider however, that these imaging studies utilized resting‐state acquisitions after NF, which differs methodologically from our active social cognition and emotionally arousing paradigm. Moreover, differences in sample composition (adults vs. adolescents) could contribute to the contradictory findings.

### 4.2 Amygdala to Cuneus Connectivity During Self‐Other Face Recognition

Depressed individuals showed higher right amygdala connectivity with the cuneus, while healthy controls demonstrated higher left amygdala connectivity (Figure 3). The cuneus (BA 17) supports visual associations and visuospatial processing (Renier et al. 2010). Occipital areas including the cuneus are also involved during the recall of autobiographical memories (Addis et al. 2004; Cabeza et al. 2004; Conway et al. 1999). The left amygdala is thought to facilitate explicit emotional functions and positive emotion (Costafreda et al. 2008; Dyck et al. 2011). Thus, it is possible that healthy youth might be explicitly regulating emotions and/or experiencing more positive emotions during self‐recognition (Costafreda et al. 2008; Renier et al. 2010). Conversely, higher right amygdala‐cuneus connectivity in depressed youth could indicate a reliance on implicit emotion regulatory strategies and/or less general positive emotions during self‐recognition compared to controls (Costafreda et al. 2008; Dyck et al. 2011; Lane et al. 2000). These findings are consistent with previous reports suggesting that a negative cognitive style may influence how self‐faces are perceived (Caudek and Monni 2013). This suggests two treatment choices; first neurofeedback training could focus on left amygdala activity and connectivity to drive patients into a typical pattern, consistent with a corrective use of neurofeedback. Alternatively, neurofeedback might target the right amygdala, paired to positive stimuli and, in a compensatory use of the training, rely on existing neural resources which depressed youth can engage. Current and ongoing research suggests that both approaches are valid and linked to positive gains in depressed and suicide‐attempting youth. For example, high right amygdala to front‐cortical connectivity was linked to lower rumination after neurofeedback, but left amygdala to frontal‐cortical connectivity was negatively correlated with depression improvement (Quevedo et al. 2020). This suggests both that depressed youth might preferentially engage implicit emotion regulation circuits and that a compensatory approach (i.e., target the right amygdala) of neurofeedback is a viable therapeutic approach that engages both the left and right amygdalae.

### 4.3 Rumination and Depression Changes and Amygdala Circuitry During Visual Self‐Recognition

Given that depressed youth had higher right amygdala to cuneus connectivity relative to controls at both time points (Figure 3), and that lower right amygdala connectivity was associated with lower rumination after scanning, it is plausible to infer that high connectivity between the right amygdala and the cuneus may contribute to the pathophysiology of depression and emotion dysregulation. Consequently, by attenuating automatic emotional responses and modulating self‐related processes, there is a potential for reducing ruminative and emotion dysregulation tendencies. This finding further supports the notion that the right amygdala‐cuneus circuitry is linked to automatic emotion dysregulation and intrusive rumination, which are characteristic of depressive states.

In contrast, depressed youth had lower connectivity between the *left* amygdala and the cuneus relative to controls (Figure 3). However, *increased left amygdala to cuneus connectivity* in depressed youth was associated with a greater reduction in depressive symptoms (i.e., less depression after vs. before NF). This finding suggests that enhancing connectivity between regions involved in effortful self‐referential and visual processing, which are typically depleted in depressed youth compared to controls, may have adaptive benefits for depressed individuals. Furthermore, the up‐regulation of left amygdala circuitry might decrease depressive symptomatology, including both cognitive and emotional symptoms. These results shed light on the differential role of connectivity between both the right and the left amygdala to the other cortical areas and their relation to depressive symptomatology. Moreover, they highlight the significance of decreasing automatic and increasing voluntary emotional to visual coordination as beneficial effects of NF intervention.

### 4.4 Amygdala Connectivity During Self‐Other Face Recognition and AMYHIPP Activity Levels During Neurofeedback Training Paired With Self‐Face Images

There is a negative association between lower right versus left amygdala‐SMTG connectivity after NF and higher AMYHIPP activity during NF training (Table 3; Figure 5). Situated at the intersection of cortical visual processing circuits known as the “what” and “where” pathways, SMTG receives poly‐sensory information from both the “what” and the “where” pathways. The STG facilitates self‐processing (Kircher et al. 2001) and is involved in spatial exploration and awareness (Karnath et al. 2001). The STG is thought to facilitate the verbalization of social situations and modulation of affective arousal via its connections to the amygdala (Kohn et al. 2014). The left amygdala is involved in explicit emotion processing and detailed affective responses (Dyck et al. 2011). The right amygdala is important for fast, global, automatic emotional processing (Dyck et al. 2011; Fenker et al. 2005), making it crucial for survival. For example, swift recognition of threats or near‐instantaneous reading of hostile emotional cues. Our results suggest that reduced implicit versus explicit affective processing of visual and spatial features facilitated by SMTG during self‐recognition (supported by the right vs. the left amygdala circuit respectively) may be linked to higher amygdala engagement and up‐regulation during NF. An explanation is that, neurofeedback training, if successful in achieving relatively high levels of bilateral AMYHIPP engagement, switches emotional processes from the right to the left amygdala‐SMTG circuit during self‐other face recognition after neurofeedback training, perhaps indicating that implicit affective processes of “what” and “where” localization take a secondary role to explicit emotion regulation sustained by the left amygdala to superior and middle temporal gyrus. While intriguing this is merely a speculative notion that ought to be tested with specific tests for spatial exploration and awareness before and after neurofeedback to confirm or refute this explanation.

A second result (Table 3) showed that higher right versus left amygdala‐middle frontal gyrus (MFG, BA9) connectivity during self‐recognition after NF training, had a trending association to higher AMYHIPP activity during NF. This is evidenced by a positive relationship between right versus left amygdala amygdala‐MFG connectivity during self‐recognition after neurofeedback and higher AMYHIPP activity during neurofeedback (Table 3; Figure 6). The right amygdala is involved in automatic emotional saliency and is predominantly associated with negative affect (Costafreda et al. 2008; Cremers et al. 2010; Dyck et al. 2011). The MFG is known to enable “top‐down” regulation of emotional processing and attention (Corbetta et al. 2008). Therefore, enhanced coordination between high right versus left amygdala to MFG connectivity after NF linked to heightened amygdala activity during NF, suggests greater control over implicit emotional responses during self‐other face recognition may have been facilitated by better NF task performance. Since depressed patients typically exhibit reduced amygdala‐MFG connectivity (Wackerhagen et al. 2020), the observed trend suggests rebalancing of this circuit might contribute to greater control over AMYHIPP activity. This explanation, again must be weighed against alternative processes and tested by measuring “top‐down” emotional control after versus before neurofeedback training.

This work expands prior findings regarding amygdala laterality and depression during and after neurofeedback training and social cognition. Specifically, Dr. Quevedo's research showed that depressed youth had greater *right amygdala* to right fronto‐cortical connectivity and *lower left amygdala* to right fronto‐cortical connectivity compared to healthy controls during neurofeedback, who showed the opposite pattern (Quevedo et al. 2020). Similar results are yielded here in a group by hemisphere interaction whereby depressed youth showed higher right amygdala to cuneus connectivity during self‐other face recognition, while healthy control youth showed higher left amygdala to cuneus connectivity (Figure 3). In this context, the links of high amygdala modulation during neurofeedback to reduced right versus left amygdala‐SMTG connectivity after neurofeedback, yet increased right versus left amygdala‐MFG connectivity after training links to high amygdala engagement during training, suggest that neurofeedback might change the relative balance of emotion regulation and social spatial processing from implicit processes (predominant in depressed individuals) to explicit control that is more characteristic of healthy populations. Again, this explanatory framework ought to be tested with instruments that probe explicit versus implicit regulatory processes in future neurofeedback research.

## 5 Limitations

Due to the lack of a placebo condition, it is difficult to discern whether rumination and depression improvements over the course of 4–5 h are the result of a score's regression toward the mean, habituation, the strategy of positive memory recall alone, or due to neurofeedback training. Also, larger sample sizes in each group could augment the power of our correlation analysis. For now, the interpretation of correlations between amygdala‐cuneus connectivity and changes in rumination and depression must be taken with caution. Including a placebo and a no‐treatment control group in future studies would improve the evidence regarding neurofeedback's efficacy and allow for conclusions regarding whether neurofeedback or other elements of the intervention are driving the effects. Additionally, PPI analyses provide two potential explanations for changes in connectivity. One possibility is that the self‐other face recognition task modulated the interaction between the amygdala seed with the reported cortical areas of connectivity. However, it is equally possible that amygdala contributions to those areas during the task modulated the responsiveness of those cortical areas regions to the task conditions (Friston et al. 1997). To distinguish between these two explanations, a comprehensive understanding of the underlying anatomical white matter tracts that facilitate connections to and from these areas is needed. Alternatively, employing dynamic causal modeling (DCM) in future research and a separate paper could help clarify the direction of connectivity influences.

We found no significant clusters connected to the left or right hippocampus, nor to the combination of hippocampus and amygdala, when jointly examined during the ESOM task or the neurofeedback training. This might suggest that hippocampal circuitry does not significantly differentiate the sampled youth during self‐versus‐other face recognition or during neurofeedback training. Alternatively, task conditions (self‐other face recognition or self‐face neurofeedback versus other‐face count‐backward) might not be different enough from each other in hippocampal connectivity measured by PPI. Lack of results for the hippocampus yet significant results for amygdala connectivity may also be due to differences in signal homogeneity within both areas and the resulting signal dis‐harmony (or harmony) in their circuitry. Signal homogeneity within an area, or similar fMRI signal fluctuations with other remote areas (e.g., with the middle frontal gyrus or the middle temporal gyrus), likely varies depending on localization within the brain and the mental activity under study. It is unlikely that the various nuclei of the hippocampus and the amygdala exhibit similar signal homogeneity, or that different nuclei are connected to the same networks. In fact, resting state fMRI connectivity studies suggest various connectivity patterns with prefrontal regions for nuclei comprising the amygdala (Bickart et al. 2012; Roy et al. 2009) and hippocampus (Cheng et al. 2020; Peng et al. 2023). Thus, it is possible that the amygdala exhibits more synchronized activity (higher internal signal homogeneity) than the hippocampus during the selected tasks, yielding false negatives for the hippocampus. We acknowledge that signal homogeneity analyses for amygdala and hippocampus nuclei (separately and as a neural complex) could further elucidate their separate and joint roles during the ESOM task. Finally, our sample consisted of predominately white females whose parents were generally married. Thus, it is unclear whether our results would generalize to a broader population of adolescents across more representative racial backgrounds and more varied family structures.

## 6 Conclusions

We sought to investigate how a short neurofeedback training, specifically, the ESOM‐NF training involving enhancement of positive self‐processing by pairing voluntary positive autobiographical memory recall to happy self‐faces, influences the bilateral amygdala circuitry engaged during self‐referential processing, operationalized as self‐other face recognition. Depressed adolescents showed a connectivity pattern of higher bilateral amygdala‐MCS circuits during self‐processing compared to healthy controls, before versus after neurofeedback training. Additionally, depressed youth showed stronger right amygdala to cuneus connectivity, during self‐other face recognition, compared to healthy controls, links between low right amygdala‐cuneus circuits and low rumination, and high left amygdala‐cuneus circuits and depression improvement in depressed youth suggest that these circuits may be an underlying mechanism via which neurofeedback may improve emotion regulation. Finally, amygdala neurofeedback appears to be a promising treatment for adolescent depression, and our research suggests that relying on existing compensatory neural mechanisms is a viable strategy of neurofeedback training for depressed youth. Some of ICCs across left and right amygdala and hippocampus yielded high agreements, but not all (Data S5). ICCs within the bilateral amygdala were consistently higher but ICCs within the bilateral hippocampus had poor agreements (Data S5). Taken together with the lack of significant circuits' engagement for hippocampus seeds added to significant results for the bilateral amygdala, these findings support that the amygdala may be a more reliable and preferable target for neurofeedback. Finally, moderate coherence between the amygdala and hippocampus (Data S5) supports neurofeedback across both amygdala and hippocampus as a joint neurological complex for neurofeedback and the ESOM task before and after neurofeedback training.

## Author Contributions

Sewon Oh: formal analysis, data curation, writing – original draft, writing – review and editing, visualization. Niki Hosseini‐Kamkar: writing – original draft, writing – review and editing. Carmen Santana‐Gonzalez: formal analysis, data curation, writing – original draft, visualization. Janani Ranatunga: writing – review and editing. Giang‐Hoang Nguyen: visualization. Matthew Maurice: writing – review and editing. Kymberly Young: writing – review and editing. Karina Quevedo: conceptualization, validation, resources, data curation, writing – review and editing, supervision, funding acquisition.

## Funding

This work was supported by grants awarded to K.Q. from the Brain and Behavior Research Foundation (NARSAD Young Investigator Award) (Grant No. UL1TR002494), and the University of Minnesota Clinical and Translational Science Institute for data collection and analysis and manuscript preparation. The content is solely the responsibility of the authors and does not necessarily represent the official views of the National Institutes of Health's National Center for Advancing Translational Sciences (UM1TR004405).

## Disclosure

CC BY Agreement: All authors agree that the article will be published under a Creative Commons Attribution (CC BY) license.

## Ethics Statement

The study was conducted in accordance with the Declaration of Helsinki and approved by the Institutional Review Board at the University of Minnesota (protocol code 1502M63041 and date of approval 26 February 2015).

## Consent

Informed consent and assent were obtained from all participants involved in the study.

## Conflicts of Interest

The authors declare no conflicts of interest.

## Supporting information


**Data S1:** jnr70097‐sup‐0001‐supinfo1.zip.

**Data S2:** jnr70097‐sup‐0002‐CREDnfChecklist.pdf.

## Data Availability

The data presented in this study is available at the request of the corresponding author. The data is not publicly available due to privacy and ethical restrictions.
