# Supplementary material for: Self‐Processing Circuits Among Depressed Youth After Amygdala Neurofeedback Cued to the Self‐Face
Source: J Neurosci Res. 2025 Dec 12;103(12):e70097. doi: 10.1002/jnr.70097 (PMC12700847; doi:10.1002/jnr.70097)
Supplement: Supplementary file 1 — Data S1: jnr70097‐sup‐0001‐supinfo1.zip. [file JNR-103-e70097-s001.zip › JNR_70097_supporting Information.docx]

**I. Psychophysiological Interaction Processing Pipeline.**

**
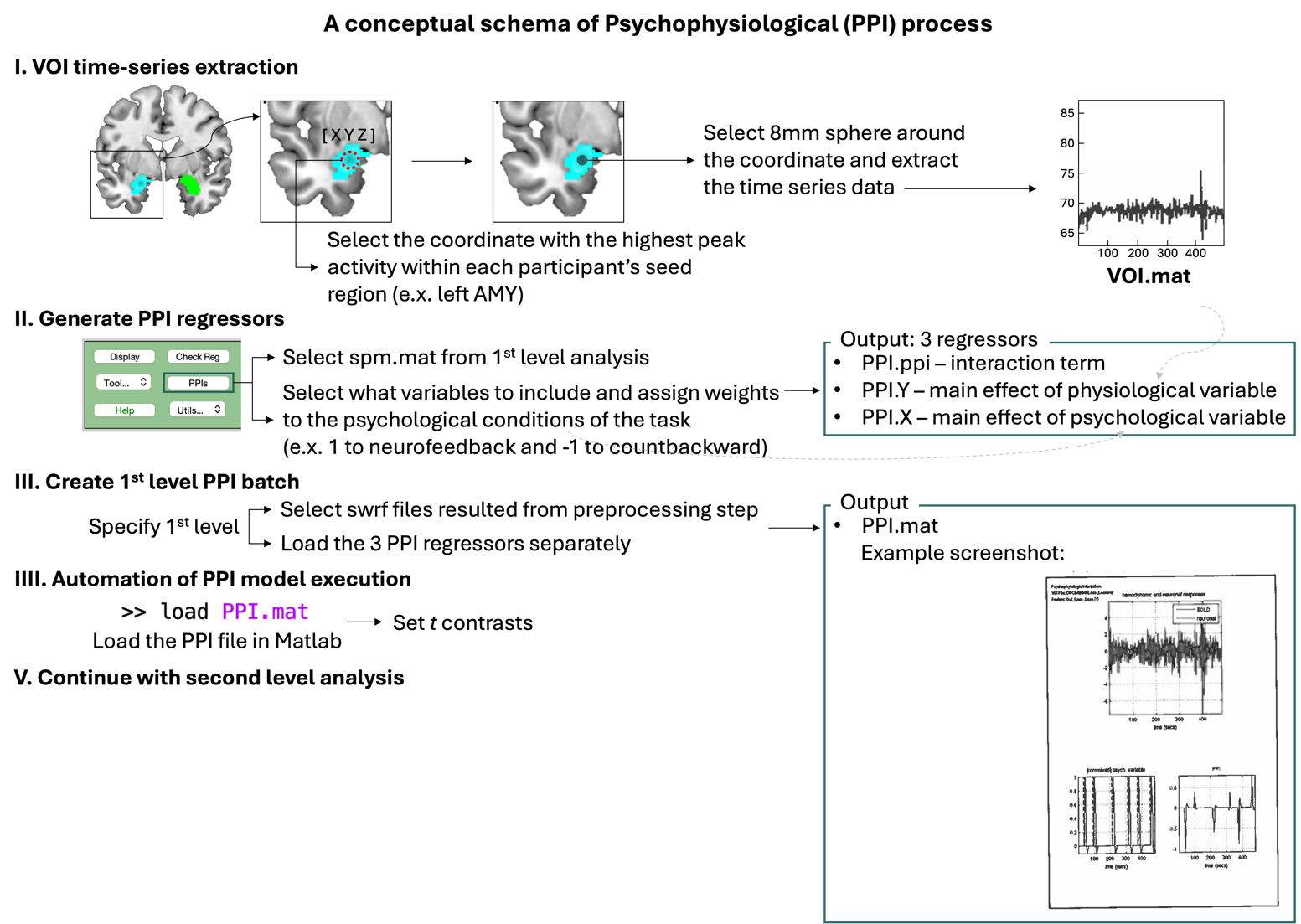
**

**Supplementary Figure 1. A Conceptual Schema of Psychophysiological (PPI) process. ‘**swrf files’ are the final output of activity analysis for each individual participant. They are the smoothed, warped, and realigned functional files for the neurofeedback task in this case.

**II. Correlation of symptoms improvements with left versus right amygdala-cuneus circuits during self-versus other face recognition.**

Estimates of amygdala connectivity reflect a group by hemisphere interaction in amygdala-cuneus connectivity. Correlation analyses between changes in symptom scores and amygdala-cuneus connectivity are conducted by each hemisphere (Supplementary Figure 2). The left amygdala connectivity did not have a significant relationship with depressive symptom reduction for neither the depressed group, *r* = 0.16, *p* = 0.375, nor the control group, *r* = 0.13, *p* = 0.581.

However, **high left amygdala-cuneus** connectivity was significantly associated to lower rumination for depressed youth, ***r* = 0.37, *p* = 0.033**. This relationship was not found in the control group, *r* = 0.23, *p* = 0.323. As the connectivity between left amygdala to cuneus strengthens after NF task, rumination symptom was reduced. The right amygdala connectivity did not have a significant relationship with depressive symptom reduction for both depressed group, *r* = -0.13, *p* = 0.472, and control groups, *r* = -0.19, *p* = 0.436. The right amygdala connectivity did not have a significant relationship with rumination symptom reduction for neither depressed, *r* = 0.04, *p* = 0.827 or control groups, *r* = 0.13, *p* = 0.588.

**
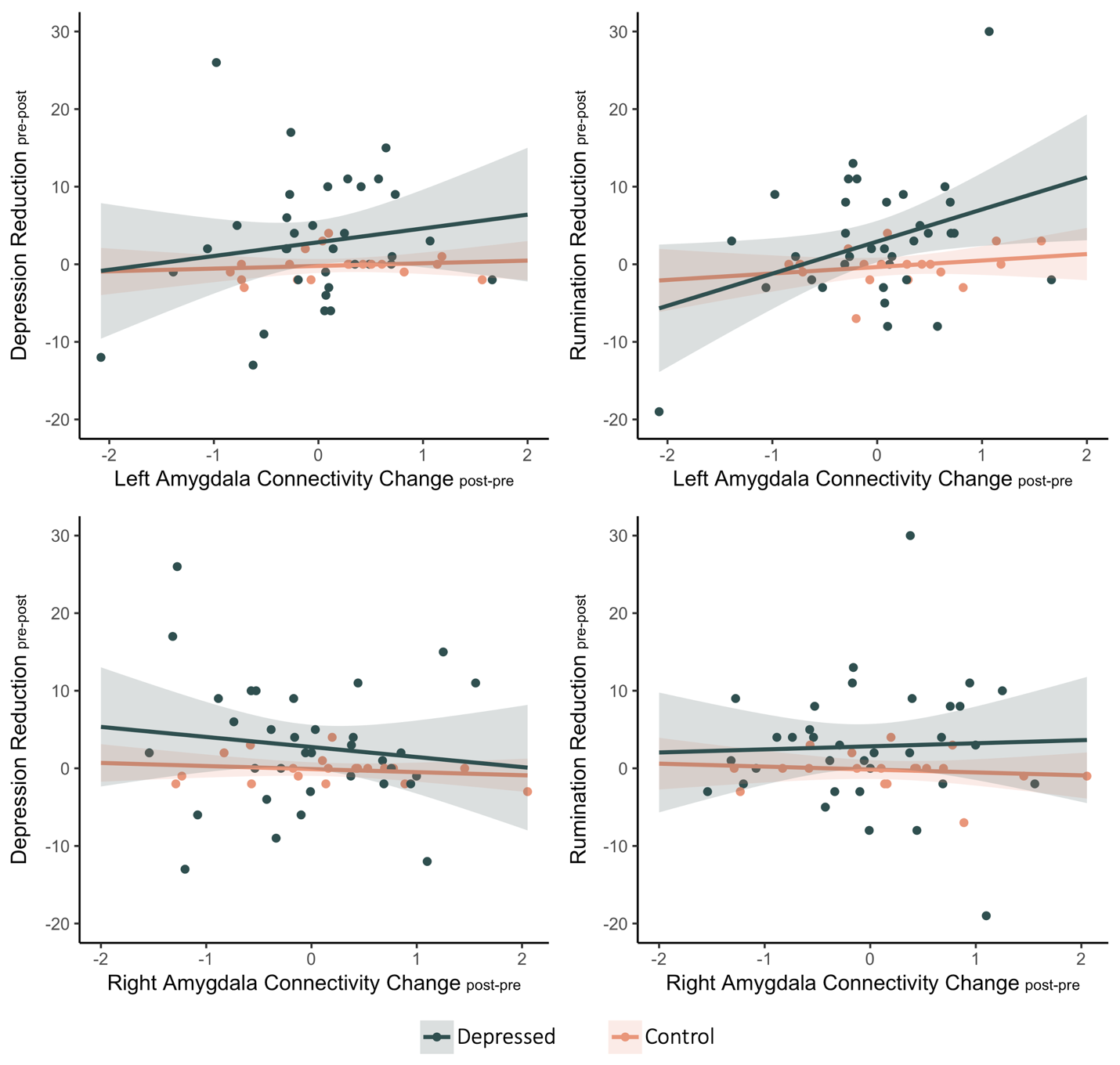
**

**Supplementary Figure 2.** Correlation between depression and rumination reduction and amygdala-cuneus connectivity change in ESOM Post-NF to Pre-NF by hemisphere. None of the relationship was significant except for the relationship between rumination change and the connectivity change. Shaded areas show 95% confidence intervals by each group.

I**II. Correlation matrix of symptom score pre- and post-NF, and their changes.**
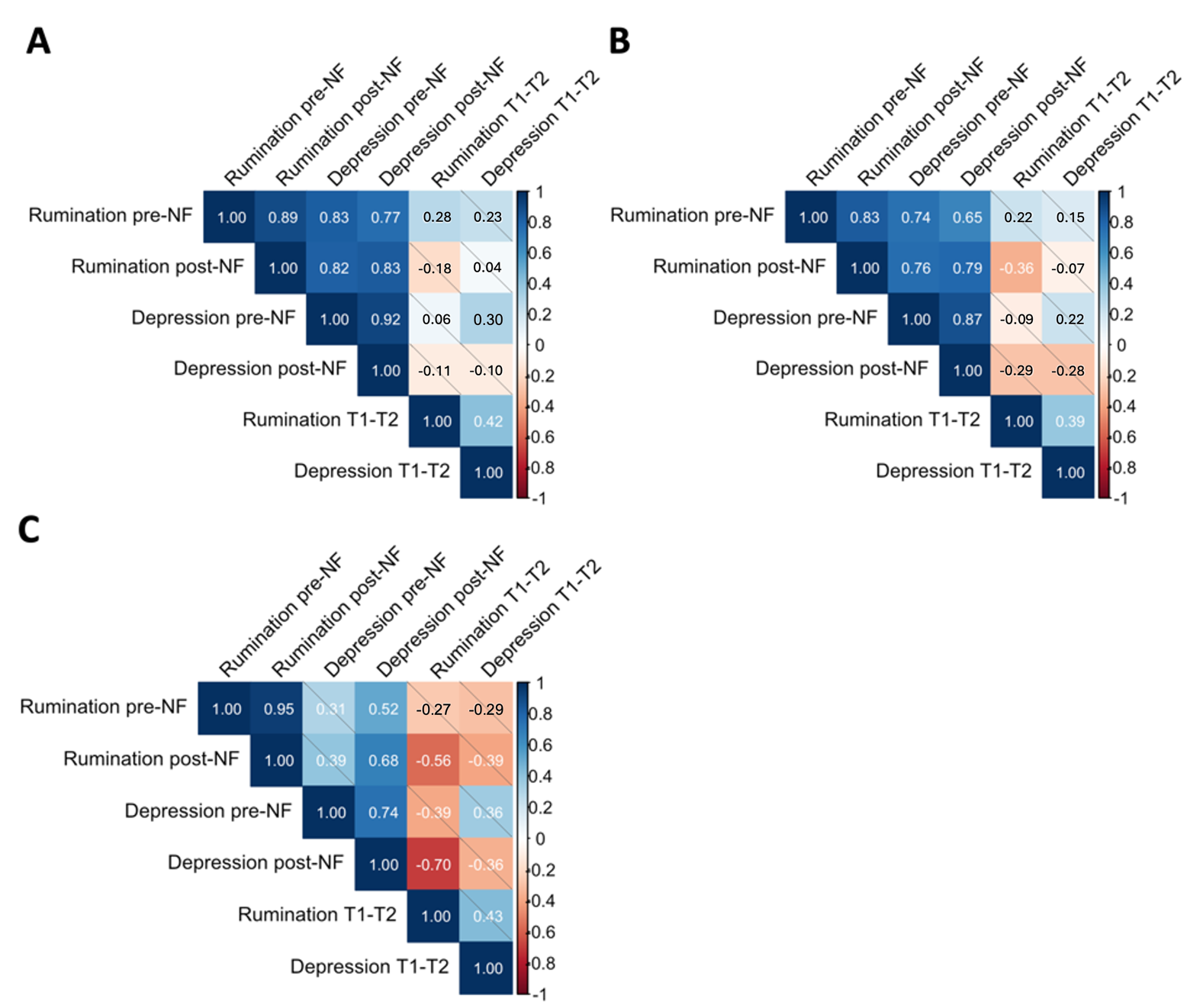


**Supplementary Figure 3**. Correlation matrix between symptom scores pre- and post-NF, and their changes from time 1 (T1) to time 2 (T2) in: **A.** all participants, **B.** within depressed youth, and **C.** within health controls. Non-significant relationships (*p* > .05) are crossed-out. Please note that the number of participants was 34 for depressed group and 19 for the healthy control group.

**IV. Time by hemisphere interaction during self vs. other face processing in ESOM task.**


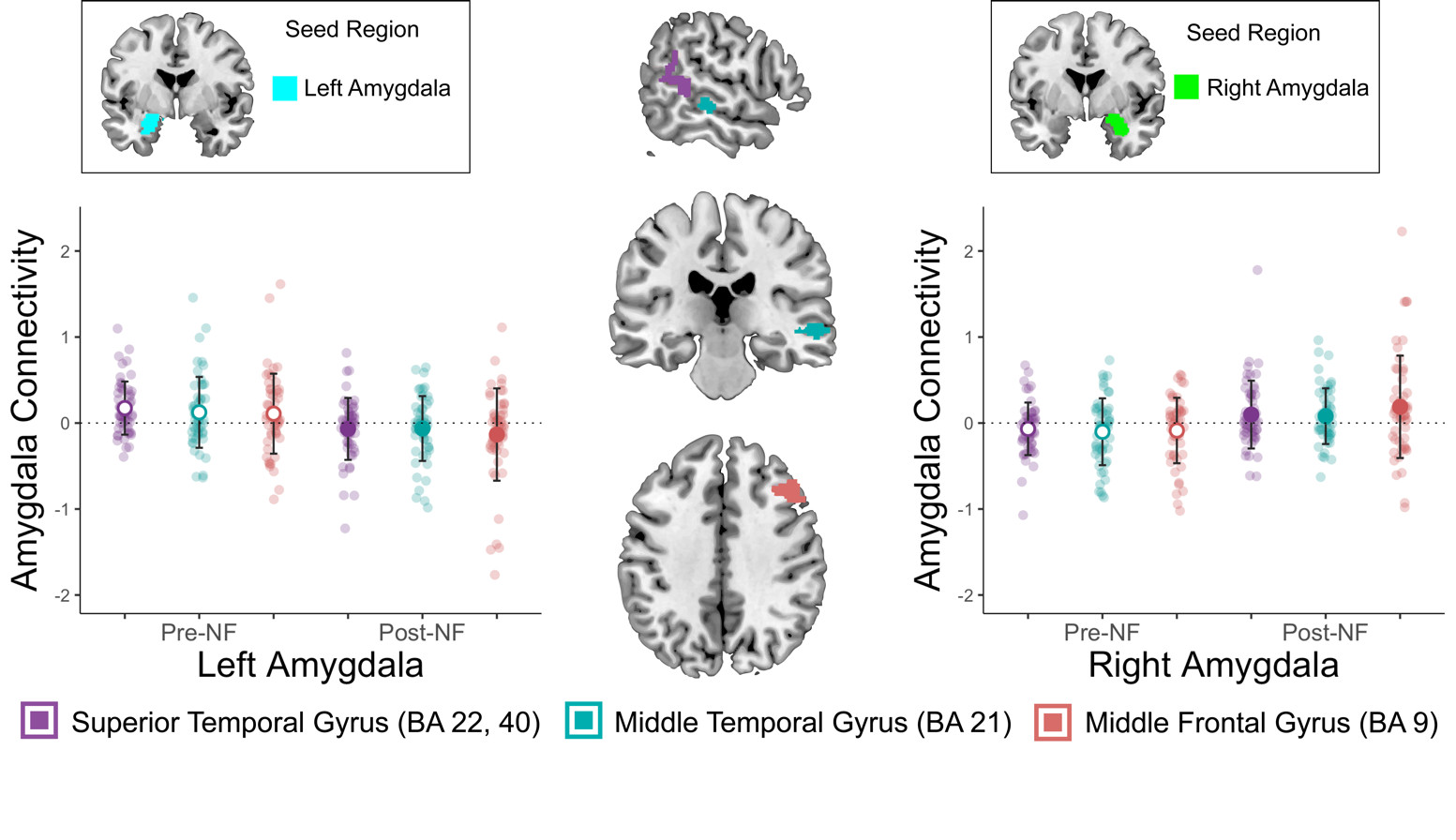


**Supplementary Figure 4. Time by Hemisphere Interaction of Amygdala Connectivity during self vs. other face recognition.** The STG, MTG and MFG (BA 21, 22, 40, 9) had different connectivity before versus after neurofeedback. Before neurofeedback there was higher left amygdala to STG, MTG and MFG connectivity during self vs other face recognition and reduced connectivity after. By contrast before neurofeedback there was low right amygdala to STG, MTG and MFG connectivity during self vs other face recognition and increased connectivity after during self vs. other face recognition. Bars represent level of connectivity with the left or right amygdala. Error bars represent ± 1 standard error.

**V. Intraclass Correlation Coefficients between left and right hemispheres within amygdala and hippocampus**

We conducted intraclass correlations (ICC) analyses across the various significant effects reported in the paper (group by time, group by hemisphere and time by hemisphere for the ESOM pre and post tasks) within the whole regions encompassed by the hippocampus left and right, within the amygdala left and right, and for the combination of amygdala and left and right, and hippocampus left and right.

ICCs are low and non-significant between the left and right hippocampus. Thus, further connectivity analyses based on subregions could elucidate if particular hippocampus subregions are driving non-significant connectivity effects.

< Hippocampus left and right >

     Group x Time for Pre: ICC = -0.294 with 95% CI [-1.268, 0.258] p = 0.819

     Group x Time for Post: ICC = 0.139 with 95% CI [-0.51, 0.506] p = 0.299

     Group x Hemi for Pre: ICC = 0.23 with 95% CI [-0.339, 0.556] p = 0.176

     Group x Hemi for Post: ICC = -0.514 with 95% CI [-1.767, 0.152] p = 0.92

     Time x Hemi for Pre: ICC = -0.0546 with 95% CI [-0.854, 0.396] p = 0.574

     Time x Hemi for Post: ICC = 0.158 with 95% CI [-0.428, 0.508] p = 0.262

Most ICCs are high and significant between the left and right amygdala. Thus, the combination of right and left amygdala can be treated as a homogenous complex in the context of ESOM and of NF.

< Amygdala left and right>

     Group x Time for Pre: ICC = **0.502** with 95% CI [0.137, 0.712] p = 0.0066**

     Group x Time for Post: ICC = **0.716** with 95% CI [0.506, 0.836] p = 6.99e-06***

     Group x Hemi for Pre: ICC = 0.201 with 95% CI [-0.4, 0.542] p = 0.214

     Group x Hemi for Post: ICC = **0.482** with 95% CI [0.115, 0.698] p = 0.00804**

     Time x Hemi for Pre: ICC = **0.451** with 95% CI [0.044, 0.684] p = 0.0171*

     Time x Hemi for Post: ICC = 0.088 with 95% CI [-0.578, 0.473] p = 0.370

Some ICCs are high and significant between the left and right amygdala and the left and right hippocampus. The other ICCs are not significant, but the associated p-values are relatively small (e.g., Time x Hemisphere Post), suggesting that the combination of right and left amygdala can be treated as a homogenous complex in the context of ESOM, particularly when sampled on the same day (i.e. pre and post time instances).

< Hippocampus and Amygdala >

     Group x Time for Pre: ICC = **0.553** with 95% CI [0.319, 0.721] p = 7.25e-05***

     Group x Time for Post: ICC = **0.415** with 95% CI [0.102, 0.637] p = 0.00692**

     Group x Hemi for Pre: ICC = 0.0132 with 95% CI [-0.513, 0.387] p = 0.462

     Group x Hemi for Post: ICC = 0.0458 with 95% CI [-0.448, 0.403] p = 0.402

     Time x Hemi for Pre: ICC = 0.164 with 95% CI [-0.28, 0.481] p = 0.203

     Time x Hemi for Post: ICC = 0.203 with 95% CI [-0.207, 0.5] p = 0.142
