# Supplementary figures and images for: Self‐Processing Circuits Among Depressed Youth After Amygdala Neurofeedback Cued to the Self‐Face
Source: J Neurosci Res. 2025 Dec 12;103(12):e70097. doi: 10.1002/jnr.70097 (PMC12700847; doi:10.1002/jnr.70097)

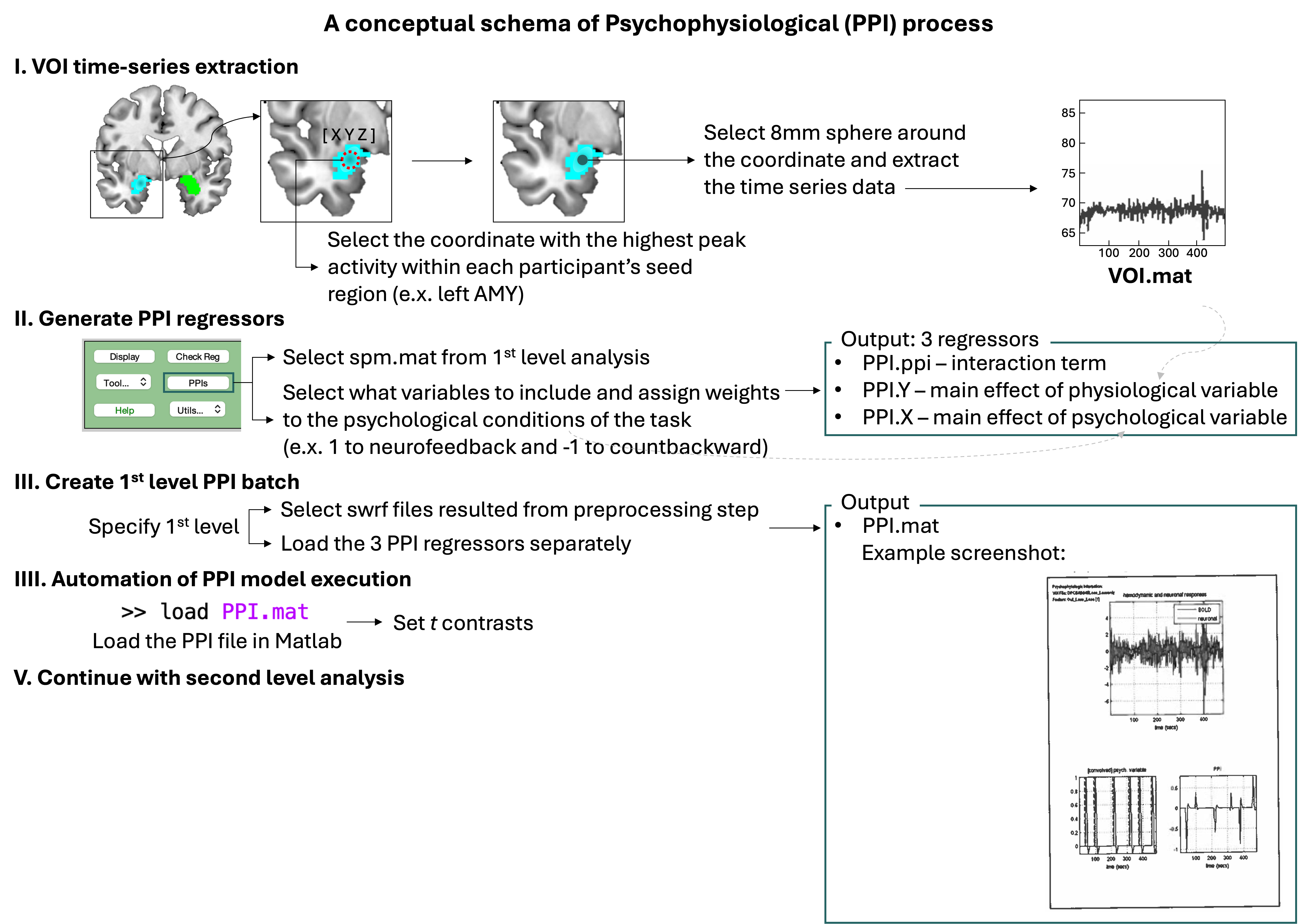

Supplement: Supplementary file 1 — Data S1: jnr70097‐sup‐0001‐supinfo1.zip. [file JNR-103-e70097-s001.zip › JNR_70097_f1_SupplFig1.png]

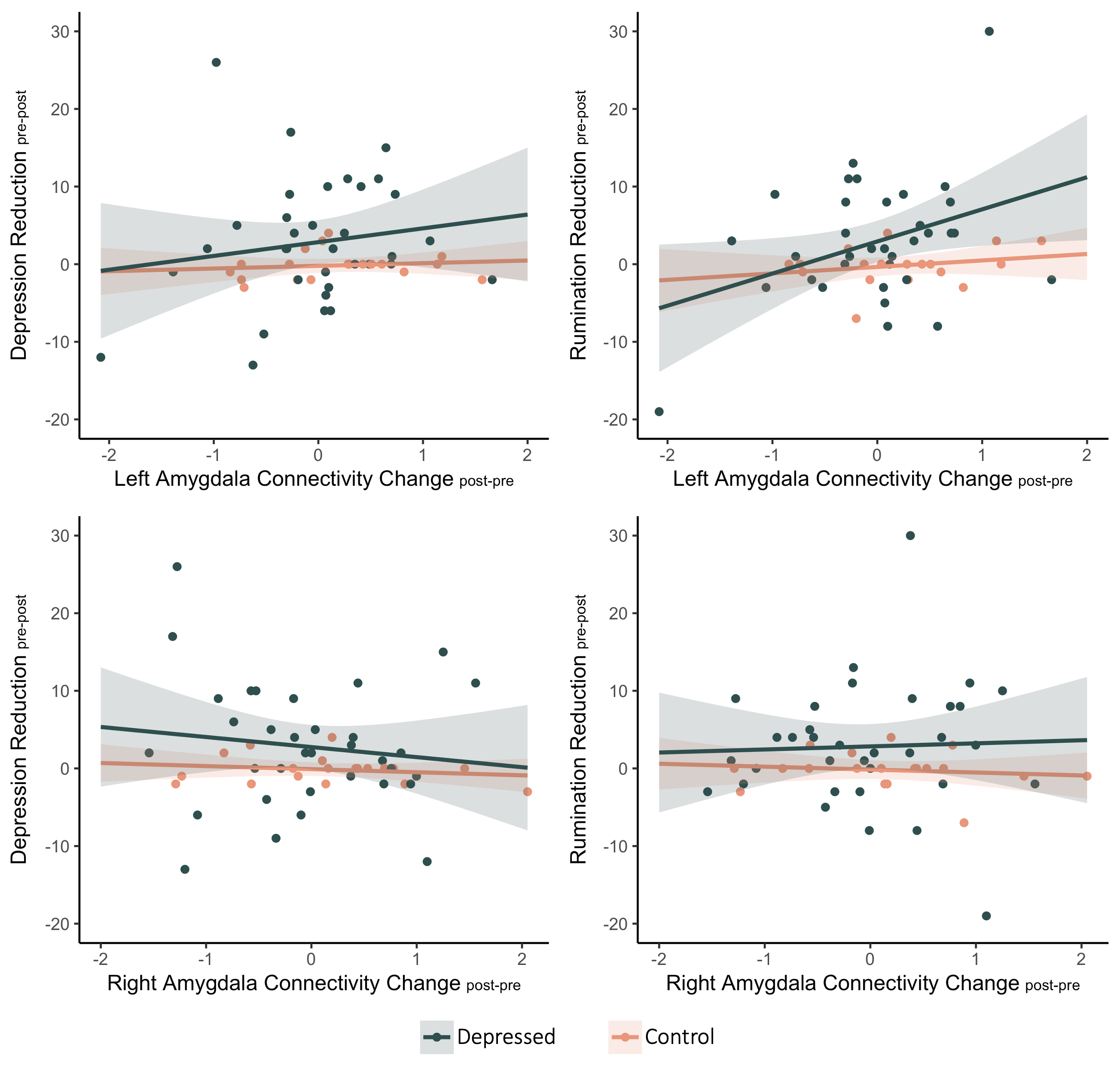

Supplement: Supplementary file 1 — Data S1: jnr70097‐sup‐0001‐supinfo1.zip. [file JNR-103-e70097-s001.zip › JNR_70097_f2_SupplFig2.png]

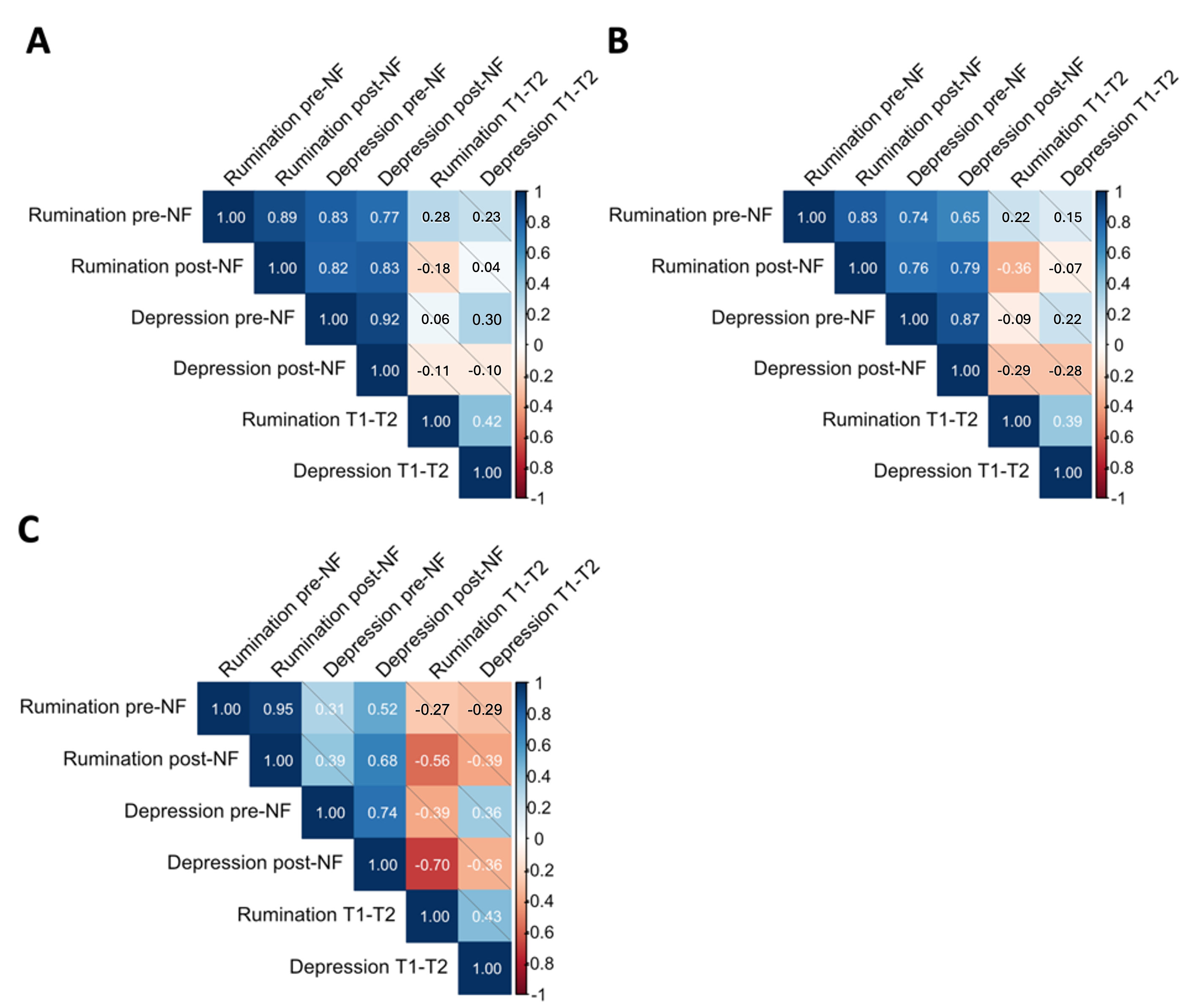

Supplement: Supplementary file 1 — Data S1: jnr70097‐sup‐0001‐supinfo1.zip. [file JNR-103-e70097-s001.zip › JNR_70097_f3_SupplFig3.png]

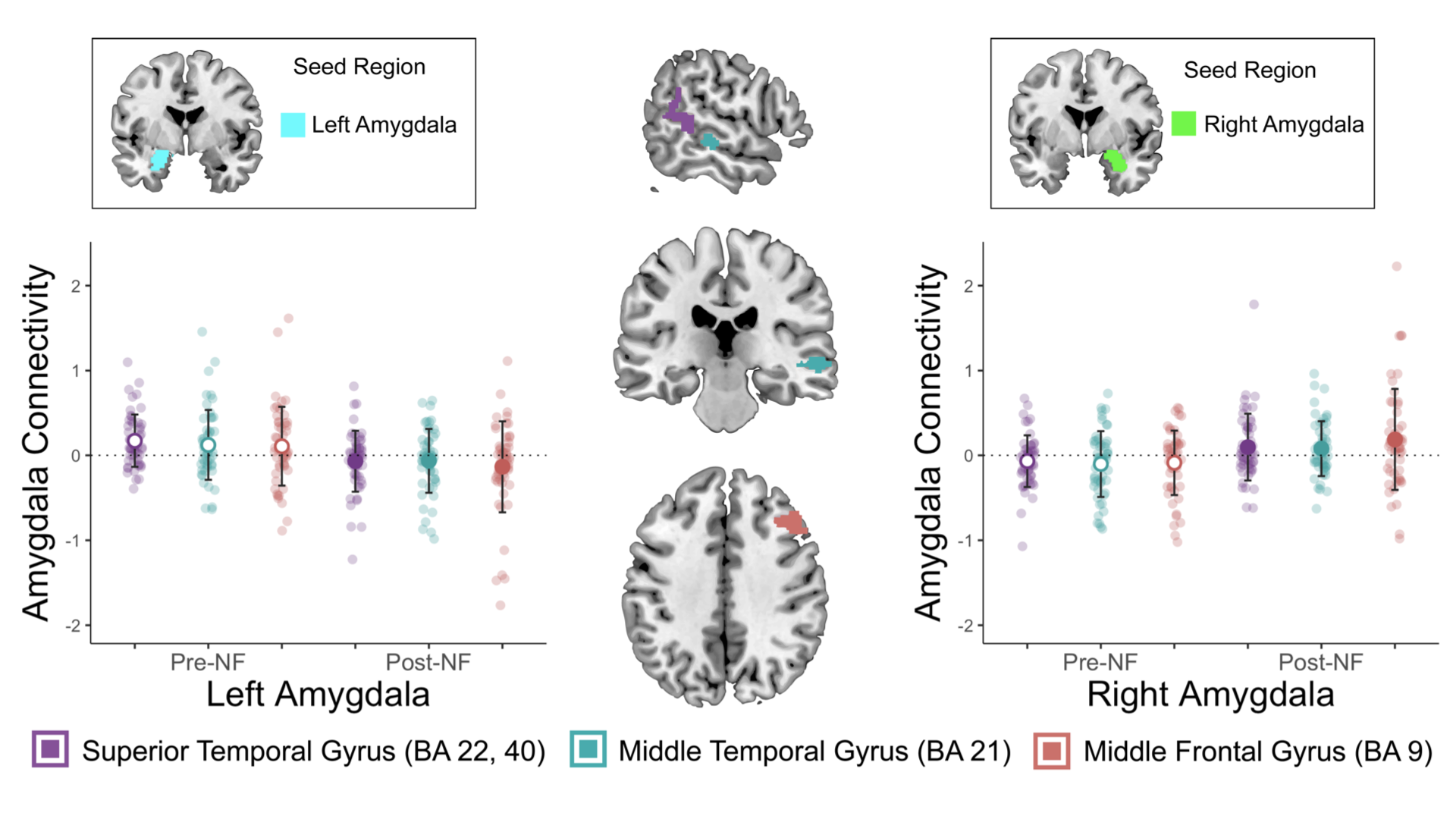

Supplement: Supplementary file 1 — Data S1: jnr70097‐sup‐0001‐supinfo1.zip. [file JNR-103-e70097-s001.zip › JNR_70097_f4_SupplFig4.tiff]
