# Supplementary material for: Self‐Processing Circuits Among Depressed Youth After Amygdala Neurofeedback Cued to the Self‐Face
Source: J Neurosci Res. 2025 Dec 12;103(12):e70097. doi: 10.1002/jnr.70097 (PMC12700847; doi:10.1002/jnr.70097)
Supplement: Supplementary file 2 — Data S2: jnr70097‐sup‐0002‐CREDnfChecklist.pdf. [file JNR-103-e70097-s002.pdf]

Consensus on the Reporting and Experimental Design of clinical and cognitive-behavioural Neurofeedback studies (CRED-nf) best practices checklist 2019*

Domain	Item #	Checklist item	Reported on page #
**Pre-experiment**			
	1a	Pre-register experimental protocol and planned analyses	NA
	1b	Justify sample size	9
**Control groups**			
	2a	Employ control group(s) or control condition(s)	9-10
	2b	When leveraging experimental designs where a double-blind is possible, use a double-blind	NA
	2c	Blind those who rate the outcomes, and when possible, the statisticians involved	NA
	2d	Examine to what extent participants and experimenters remain blinded	NA
	2e	In clinical efficacy studies, employ a standard-of-care intervention group as a benchmark for improvement	NA
**Control measures**			
	3a	Collect data on psychosocial factors	Table 1
	3b	Report whether participants were provided with a strategy	9-12
	3c	Report the strategies participants used	10-12
	3d	Report methods used for online-data processing and artifact correction	12-14
	3e	Report condition and group effects for artifacts	NA
**Feedback specifications**			
	4a	Report how the online-feature extraction was defined	12-13
	4b	Report and justify the reinforcement schedule	10-11
	4c	Report the feedback modality and content	10-12
	4d	Collect and report all brain activity variable(s) and/or contrasts used for feedback, as displayed to experimental participants	10-13
	4e	Report the hardware and software used	11-14
**Outcome measures**			
Brain	5a	Report neurofeedback regulation success based on the feedback signal	Quevedo, 2019
	5b	Plot within-session and between-session regulation blocks of feedback variable(s), as well as pre-to-post resting baselines or contrasts	Quevedo, 2019
	5c	Statistically compare the experimental condition/group to the control condition(s)/group(s) (not only each group to baseline measures)	14-21
Behaviour	6a	Include measures of clinical or behavioural significance, defined a priori, and describe whether they were reached	18-21, Table 1
	6b	Run correlational analyses between regulation success and behavioural outcomes	Figure 4
**Data storage**			
	7a	Upload all materials, analysis scripts, code, and raw data used for analyses, as well as final values, to an open access data repository, when feasible	NA

*Darker shaded boxes represent *Essential* checklist items; lightly shaded boxes represent *Encouraged* checklist items. If a checklist item is “not addressed” in the manuscript, enter “N” in place of the page number. We recommend using this checklist in conjunction with the CRED-nf article, which explains the motivation behind this checklist and provides details regarding many of the checklist items.
